# Cetyl All-*Trans*-Retinoate as a Lipidic ATRA Prodrug with Enhanced Anticancer and Chemosensitizing Activity

**DOI:** 10.3390/ijms27135982

**Published:** 2026-07-03

**Authors:** Paweł Moroz, Klaudia Muciek, Marta Świtalska, Joanna Wietrzyk, Zbigniew Lazar, Anna Gliszczyńska

**Affiliations:** 1 Department of Food Chemistry and Biocatalysis, Wrocław University of Environmental and Life Sciences, Norwida 25, 50-375 Wrocław, Poland; pawel.moroz@upwr.edu.pl (P.M.); 114975@student.upwr.edu.pl (K.M.); 2 Department of Experimental Oncology, Hirszfeld Institute of Immunology and Experimental Therapy, Polish Academy of Sciences, Weigla 12, 53-114 Wrocław, Poland; marta.switalska@hirszfeld.pl (M.Ś.); joanna.wietrzyk@hirszfeld.pl (J.W.); 3 Department of Biotechnology and Food Microbiology, Wrocław University of Environmental and Life Sciences, Chelmonskiego 37, 51-630 Wrocław, Poland; zbigniew.lazar@upwr.edu.pl

**Keywords:** all-*trans*-retinoic acid, tretinoin, anticancer activity, prodrugs, in vitro cytotoxicity, lipid conjugates

## Abstract

Tretinoin (all-*trans*-retinoic acid, ATRA) is an established therapy for acute promyelocytic leukemia (APL) and neuroblastoma (NB); however, its broader oncological application is limited by poor bioavailability and rapid resistance development. In this study, we developed lipidic ester derivatives of ATRA as a potential prodrug approach aimed at modulating its physicochemical and biological properties. Three ATRA derivatives were evaluated in vitro in six human cancer cell lines: leukemia (MV4-11), gastric (AGS), colon (HT-29), lung (A549), and breast cancer cells (MCF-7, MDA-MB-468). Cytotoxicity toward normal human breast epithelial cells (MCF-10A) was also assessed. Among the synthesized derivatives, cetyl all-*trans*-retinoate (ATRA-CA) exhibited the strongest anticancer activity, showing up to threefold greater potency than ATRA, with inhibitory concentrations ranging from 1.34 to 23.1 µM and minimal toxicity toward normal cells. Moreover, ATRA-CA enhanced the efficacy of conventional chemotherapeutics. In A549 cells, treatment with 5 and 10 µM ATRA-CA reduced the cisplatin IC_50_ from 25.7 ± 3.2 µM to 9.1 ± 3.0 and 5.9 ± 1.5 µM, corresponding to synergistic (CI = 0.63) and additive (CI = 0.88) effects, respectively. Similar effects were observed in MCF-7 cells and in combination with doxorubicin and paclitaxel.

## 1. Introduction

Tretinoin (all-*trans*-retinoic acid, ATRA) is considered the most pharmacologically active metabolite of vitamin A, playing a key role in regulating immune responses, inflammatory pathways, and cellular differentiation. It also exhibits well-documented chemopreventive and anticancer properties [1,2]. The biological activity of retinoids is primarily mediated through their interaction with nuclear receptors, retinoic acid receptors (RARs) and retinoid X receptors (RXRs), which function as ligand-activated transcription factors controlling the expression of genes involved in cell proliferation, apoptosis, differentiation, and immune regulation [3].

Structure–activity relationship (SAR) studies have identified the free carboxyl group and conjugated polyene chain of ATRA as critical determinants of retinoid receptor binding and transcriptional regulation [4]. The biological activity of vitamin A derivatives is strongly influenced by their oxidation state and chemical structure. Among naturally occurring retinoids, ATRA exhibits the highest direct activity owing to its strong affinity for retinoic acid receptors (RARs), whereas retinal, retinol and retinyl acetate as well as retinyl palmitate require enzymatic conversion to retinoic acid before becoming biologically active [5]. These differences are associated with variations in receptor affinity, metabolic activation, and compound stability.

Tretinoin has found clinical application in the treatment of acute promyelocytic leukemia (APL). The drug was introduced to the market in 1996 by Roche and is particularly recommended for patients with the characteristic chromosomal translocation t(15;17)(q22;q21), which results in the formation of the PML-RARα fusion gene [6]. This oncogene fusion protein disrupts normal promyelocyte differentiation by acting as a dominant transcriptional repressor. ATRA counteracts this effect by promoting degradation of the PML-RARα oncoprotein and restoring terminal granulocytic differentiation [7]. Tretinoin is administered orally, typically as 10 mg capsules (e.g., Vesanoid^®^). ATRA-based therapy has become the standard of care in APL and is frequently combined with arsenic trioxide (ATO) or anthracyclines. This therapeutic strategy has markedly improved complete remission rates and overall patient survival [8,9]. The effectiveness of tretinoin has transformed APL from a previously highly fatal hematologic malignancy into one of the most curable form of acute leukemia, with high rates of durable remission [10].

ATRA is also used clinically in the treatment of neuroblastoma in children, primarily as an adjuvant therapy in advanced or residual forms of the disease, particularly following chemotherapy [11]. Numerous studies have identified this drug as a potential anticancer agent across a broad spectrum of solid tumors, including cancers of the head and neck, breast, prostate, lung, ovary and gastrointestinal tract [12,13,14,15,16]. Despite promising preclinical and early clinical findings [17,18,19], ATRA has not been approved for the treatment of solid tumors. This limitation is largely attributable to its significant toxicity profile and the rapid emergence of therapeutic resistance [17,20]. A substantial proportion of patients may develop differentiation syndrome, also known as retinoic acid syndrome (RAS), a potentially life-threatening complication characterized by fever, respiratory distress, weight gain, hypertension and, in severe cases, acute renal failure [21]. Resistance to ATRA may arise during therapy, most commonly due to enhanced metabolic degradation, resulting in a rapid decline in plasma drug concentrations.

One strategy to overcome the physicochemical limitations of ATRA [22], reduce its toxicity, and improve its biological properties involves structural modification of the parent molecule. Various approaches have been explored, including the development of synthetic retinoid analogues such as AM580, arotinoids, and heteroarotinoids. Findings presented by Abdelaal and co-workers [23] suggest that selected retinoic acid derivatives may exhibit stronger and more sustained antitumor activity compared with the unmodified parent compound.

In parallel, formulation-based strategies aimed at improving the therapeutic performance of ATRA have also been extensively investigated. In particular, nanotechnology-based approaches involving the encapsulation of ATRA into carrier systems, including nanoparticles, liposomes, and microspheres, have been proposed to enhance its pharmacokinetic profile, improve delivery to target tissues, and reduce systemic toxicity. Giuli et al. [24] evaluated various carrier systems as strategies to optimize ATRA delivery and improve its therapeutic applicability. Despite intensive research efforts, none of these approaches has yet resulted in the approval of new clinical indications for tretinoin.

In this study, we explored esterification as a potential strategy to modulate the physicochemical and biological properties of ATRA and to reduce its toxicity. Temporary masking of the carboxyl group through ester bond formation may increase lipophilicity and promote cellular uptake. Moreover, ester bonds susceptible to enzymatic cleavage may enable intracellular regeneration of active ATRA under physiological conditions. Such lipidic derivatives may therefore offer an opportunity to optimize retinoid delivery, reduce non-specific toxicity, and improve the therapeutic profile of ATRA. Accordingly, the present proof-of-concept study aimed to synthesize ATRA esters and to evaluate their preliminary physicochemical properties, antiproliferative activity, and selectivity toward selected human cancer cell lines.

## 2. Results and Discussion

### 2.1. Synthesis of Esters of All-Trans-Retinoic Acid (ATRA)

**Figure 1:**
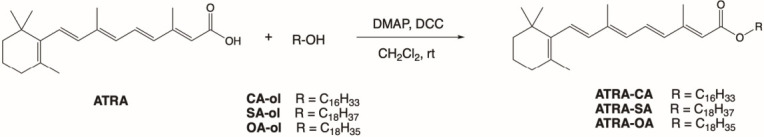
Scheme of chemical synthesis of ester derivatives of ATRA.

All-*trans*-retinoic acid was esterified with selected long-chain fatty alcohols including cetyl alcohol (CA-ol), stearyl alcohol (SA-ol) and oleyl alcohol (OA-ol) to obtain lipophilic derivatives with potential pharmaceutical applications (Figure 1). These compounds were designed as potential ester-based prodrugs that may undergo enzymatic hydrolysis by endogenous carboxylesterases, leading to the release of the parent ATRA. Such enzymatically cleavable ester linkages promote sustained release of the active compound and thereby influence its pharmacological profile [25,26].

Esters of retinoic acid: cetyl-all-*trans*-retinate (ATRA-CA), stearyl-all-*trans*-retinate (ATRA-SA) and oleyl-all-*trans*-retinate (ATRA-OA) were synthesized via an esterification reaction following the procedure previously reported by Clausen’s research group [27]. According to their findings for the reaction of acylation of ATRA, we used *N*,*N*′-dicyclohexylcarbodiimide (DCC) as a coupling agent which does not result in the contaminant isomerization [27]. The catalyst in this reaction was 4-(*N*,*N*-dimethylamino)pyridine (DMAP). The reactions were performed for 24 h at room temperature in the dark on the magnetic stirrer. After this, the products were purified. Cetyl-all-*trans*-retinate (ATRA-CA), stearyl-all-*trans*-retinate (ATRA-SA) and oleyl-all-*trans*-retinate (ATRA-OA) were obtained in good yields ranging from 58 to 75%. ATRA-CA has been previously reported in the literature [27], whereas the two remaining derivatives ATRA-SA and ATRA-OA are described here for the first time. The structures of all synthesized compounds were confirmed by nuclear magnetic resonance (NMR) (^1^H, ^13^C) and correlation spectroscopy (COSY, HSQC) (spectra included in Supplementary Materials). All characteristic signals corresponding to the all-*trans* double bond system were identified in the ^1^H NMR spectra of products, indicating that no isomerization or reduction of the double bond occurred during reaction.

### 2.2. Prediction of Physicochemical Properties and Biological Activity

Computational chemoinformatics methods were applied to analyze the physicochemical properties of the synthesized retinoic acid derivatives. The calculated descriptors included thermodynamic parameters, such as the octanol-water partition coefficient (logP), as well as structural descriptors, including the topological polar surface area (TPSA), molecular surface area, volume, and ovality. These data are summarized in Table 1. The calculated logP values indicate a marked increase in lipophilicity for the ester derivatives compared to unmodified ATRA, which may enhance passive diffusion across cell membranes and enhance their absorption in biological systems. The TPSA values [Å^2^] remain within ranges generally confirming good membrane permeability of these derivatives, suggesting favorable oral bioavailability and potential ability to cross the blood–brain barrier (BBB).

To estimate the biological activity of the ATRA derivatives, the PASS (Prediction of Activity Spectra for Substance) method was employed. Using a structure–activity relationship (SAR) approach, the P_a_ factor (probability of being active) and P_i_ factor (probability of being inactive) were calculated and analyzed for the synthesized ATRA esters. The results indicate that all esters exhibit a high potential for anticancer activity, with a probability greater than 70%, suggesting that these compounds may serve as promising candidates for the development of new anticancer agents. Table 2 summarizes the predicted biological activities and potential mechanisms of action of the synthesized compounds. The data obtained using the PASS method support the hypothesis that esterification of ATRA represents a viable strategy for the design of novel therapeutics, potentially offering improved pharmacokinetic properties and reduced side effects compared with free retinoic acid. This structural modification may improve drug bioavailability, which is crucial for clinical applications. Based on these promising predictions, further biological experiments were conducted to evaluate the antiproliferative activity of the synthesized esters against selected human cancer cell lines to validate their therapeutic potential.

In the next stage, an ADMET analysis of the synthesized prodrugs was conducted using the pkCSM predictive tool, which provided significant insights into their pharmacokinetics and safety profiles. The in silico analysis results indicate that all three esters ATRA-CA, ATRA-SA, and ATRA-OA exhibit good permeability through Caco-2 cells (log Papp ≈ 1.38) and high intestinal absorption (~89%), suggesting favorable oral bioavailability (Table 3). Importantly, none of the compounds were identified as P-glycoprotein substrates; however, all of them act as P-glycoprotein type II inhibitors. This may influence their drug–drug interaction profiles, particularly with regard to active transport across cell membranes. All esters also demonstrated a low volume of distribution (log VDss close to 0), which suggests limited penetration into peripheral tissues. Regarding their ability to cross the blood–brain barrier (log BB > 0.88), these compounds may potentially exert effects on the central nervous system. However, their low log PS values (below −1.1) indicate limited actual penetration into the CNS [26]. In terms of metabolism, only ATRA-CA exhibited CYP2D6 inhibitory activity, which could be clinically relevant in the case of co-administration with drugs metabolized by this enzyme. All analyzed esters, however, are metabolized by CYP3A4, the main hepatic enzyme responsible for the biotransformation of many active pharmaceutical ingredients. The esters also showed similar rates of total clearance, and the lack of affinity for the renal organic cation transporter (OTC) suggests they are unlikely to undergo active renal excretion. Regarding safety, none of the esters showed mutagenic activity in the AMES test. These findings confirm that ATRA esters exhibit promising pharmacokinetic properties and favorable safety profiles, making them valuable candidates for further preclinical studies. Therefore, in the following stage, we conducted a series of in vitro activity assays using selected cancer cell lines.

### 2.3. Cytotoxicity of ATRA and Its Esters Towards Selected Cell Lines

Retinoic acid and its lipid derivatives ATRA-CA, ATRA-OA and ATRA-SA were evaluated for their antiproliferative activity in the in vitro model against six human cancer cell lines representing malignancies with high clinical burden. The panel included leukemia (MV4-11), lung cancer (A549), colon cancer (HT-29), gastric cancer (AGS), triple-negative breast cancer (MDA-MB-468, ER^-^, PR^-^, HER2^-^, mutant p53) and hormone receptor-positive breast cancer (MCF-7, ER^+^, PR^+^ glucocorticoid receptor-positive). The panel included both hematological and epithelial cancers in which retinoid signaling has been implicated in the regulation of proliferation, differentiation and apoptosis. The use of triple-negative breast cancer (MDA-MB-468) and hormone receptor-positive breast cancer (MCF-7) models enabled comparative evaluation of retinoid activity in molecularly distinct breast cancer subtypes characterized by different therapeutic sensitivities and receptor profiles. As a control we evaluated also the antiproliferative activities of fatty alcohols: cetyl alcohol (CA-ol), oleyl alcohol (OA-ol) and stearyl alcohol (SA-ol). Possible cell toxicity of esters was tested by the rate of viability of the human normal breast epithelial MCF-10A cells and compounds were tested at concentrations ranging from 0.2 to 633 µM with a 72 h incubation period.

Among all tested compounds, only cetyl-all-*trans*-retinate (ATRA-CA) demonstrated significantly higher anticancer activity than retinoic acid with IC_50_ values ranging from 1.34 to 23.1 µM. Oleyl-all-*trans*-retinate (ATRA-OA) was less active than ATRA-CA and inhibited the proliferation of studied cancer cells at higher concentrations (30.94–102.4 µM). The least active turned out to be a derivative of stearic acid ATRA-SA which reduced the growth of cancer cells at a much higher concentration (112.73–496.80 µM) than the previous two esters. All results obtained are summarized in Table 4.

The observed nonlinear relationship between the biological activity of the synthesized ATRA prodrugs and alkyl chain length is consistent with numerous reports in the literature. It has been demonstrated that the biological activity of esters and lipid derivatives does not increase monotonically with rising lipophilicity, rather, it reaches a maximum at a specific “optimal” alkyl chain length and then declines sharply. This phenomenon, known as the “cut-off effect”, is a well-established feature of structure–activity relationships (SAR/QSAR). Studies on phenolic esters, phenolipids, and other amphiphilic bioactive compounds have shown that extending the alkyl chain by only one or two carbon atoms beyond the optimum can result in a significant reduction in biological activity, despite very similar physicochemical properties [28]. This effect is commonly attributed to a disruption of the balance between membrane penetration capacity and efficient intracellular distribution, solubility, and accessibility to the molecular target [29].

Free ATRA which is clinically used in the treatment of acute myeloid leukemia (AML) [30,31,32,33] was identified as the most active compound against leukemia cells among the tested agents. In MV4-11 cells, ATRA inhibited proliferation with an IC_50_ value of 1.08 µM. Obtained results are in accordance with that reported for free ATRA in the literature [34,35]. Esterification of ATRA with cetyl alcohol did not substantially reduce its activity. The half maximal inhibitory concentration (IC_50_) of ATRA-CA was comparable to that of free ATRA and was determined to be 1.34 µM. In contrast, esterification with oleyl alcohol resulted in approximately 30-fold lower activity, whereas ATRA-SA exhibited markedly reduced potency, inhibiting leukemia cell proliferation only at significantly higher concentrations (IC_50_ = 112.73 µM). Our data indicate that ATRA-CA also exhibits strong antiproliferative activity against human lung carcinoma A549 cells, with an IC_50_ value of 12.98 µM, demonstrating slightly greater potency than free ATRA (IC_50_ = 14.14 µM). As shown in Table 4 esterification of retinoic acid with cetyl alcohol resulted in enhanced antiproliferative activity compared with the parent compound in two additional tumor cell lines. ATRA-CA demonstrated higher cytotoxicity against the HT-29 cell line (IC_50_ = 8.5 µM) and AGS cell line (IC_50_ = 8.78 µM) than free retinoic acid which was active at concentrations 11.05 and 21.8 µM, respectively. It is worth to mention that obtained results of activity of free ATRA against colon cancer and gastric cancer are similar to those reported by Osmond [36] and Patrad [37] for the same type of cancer. The anticancer activity of ATRA in breast cancer has been widely documented [38,39,40,41]. In our study, the most pronounced differences between free ATRA and its ester derivatives were observed in breast cancer models. While free ATRA inhibited the proliferation of triple-negative breast cancer (MDA-MB-468) and hormone receptor-positive MCF-7 cells with IC_50_ values of 78.2 and 22.03 µM, respectively, ATRA-CA exhibited 3.4- and 2.6-fold higher activity (IC_50_ = 23.1 and 8.34 µM, respectively).

The obtained results clearly demonstrate substantial variability in the sensitivity of the tested cell lines to ATRA and its ester derivatives. The pronounced sensitivity of MV4-11 is consistent with the well-established efficacy of retinoids in hematologic malignancies, where activation of retinoic acid receptors (RARs) induces differentiation of malignant cells [42,43]. In contrast, the solid tumor cell lines (A549, HT-29, AGS, MDA-MB-468, and MCF-7) exhibited significantly higher IC_50_ values, suggesting reduced sensitivity to retinoid treatment in these models [44]. The observed differences may result from variable expression levels of nuclear receptors RAR/RXR, which mediate the biological activity of ATRA, as well as from differences in the activity of enzymes involved in retinoid metabolism. The distinct molecular profiles of the analyzed cell lines may also play a significant role, including differences in hormone receptor status, mutations in tumor suppressor genes and oncogenes, and the activity of key proliferative signaling pathways [45,46,47]. Additionally, differences in activity may stem from variable capacities of individual cell lines to hydrolyze the esters and release free ATRA. Variations in proliferation rate, receptor status, and genetic background may further modulate cellular sensitivity to compounds that act predominantly through cytostatic mechanisms.

In the evaluation of the anticancer potential of novel compounds, it is essential not only to determine their cytotoxic effects against cancer cells but also to assess the selectivity of their action. Ideally, antiproliferative activity should preferentially target malignant cells while sparing non-tumorigenic cells. Therefore, the human mammary epithelial cell line MCF-10A, a non-tumorigenic model, was used as a control to evaluate compound selectivity. The in vitro effects of the tested compounds on the MCF-10A cells were used to calculate the selectivity index (SI). The SI was determined by dividing the IC_50_ value determined for the normal MCF-10A cell line by the IC_50_ value determined for each respective cancer cell line. The results are presented below in Table 5.

SI values below 1.0 indicate a lack of selectivity and suggest general cytotoxicity, whereas values greater than 1.0 reflect preferential activity towards cancer cells relative to non-tumorigenic cells. Higher SI values correspond to greater selectivity and may indicate a potentially improved therapeutic window. Notably, ATRA-CA, the most active derivative against leukemia cells, was characterized by a very high SI value of 54.03. This value was higher than the SI determined for the parent compound ATRA, indicating a favorable selectivity profile for ATRA-CA. This compound also showed good selectivity toward lung, colon, gastric and MCF-7 breast cancer cells. Analysis of the obtained results in relation to the chemical structure of the synthesized derivatives suggests that both the length of the attached aliphatic chain and its degree of saturation may influence antiproliferative activity and selectivity.

From a structural perspective, the more favorable selectivity profile of ATRA-CA may be associated with an optimal balance between the retinoid pharmacophore and the attached saturated C16 alkyl chain. Such a structural arrangement appears to provide sufficient lipophilicity to promote interactions with cellular membranes and cellular uptake, while avoiding excessive hydrophobicity that could limit intracellular distribution or reduce the accessibility of the compound to enzymatic hydrolysis and molecular targets. In contrast, further elongation of the alkyl chain, as in ATRA-SA, or the introduction of a double bond, as in ATRA-OA, may disturb this balance. This interpretation is consistent with the nonlinear relationship between alkyl chain length and biological activity observed for amphiphilic lipid derivatives, commonly referred to as the “cut-off effect” [28,29]. This structure-dependent relationship is particularly evident from the SI values calculated for ATRA-CA, which were higher than those obtained for the parent compound ATRA in several solid tumor models, including HT-29, AGS, MDA-MB-468, and MCF-7 cells.

The analysis of the hydrolytic stability of the most active prodrug ATRA-CA demonstrated that the compound remains stable at pH 7.2, whereas under acidic conditions (pH 1.2), it undergoes hydrolysis (See Section 3.4). The content of ATRA-CA gradually decreased with increasing incubation time, reaching complete hydrolysis before 24 h (Supplementary Materials Figure S15). These findings suggest that ATRA-CA exhibits stability under physiological pH conditions while remaining susceptible to acid-catalyzed hydrolysis under acidic conditions.

### 2.4. Cell Cycle Analysis of Lung Cancer Cells After Incubation with ATRA and ATRA-CA

**Figure 2:**
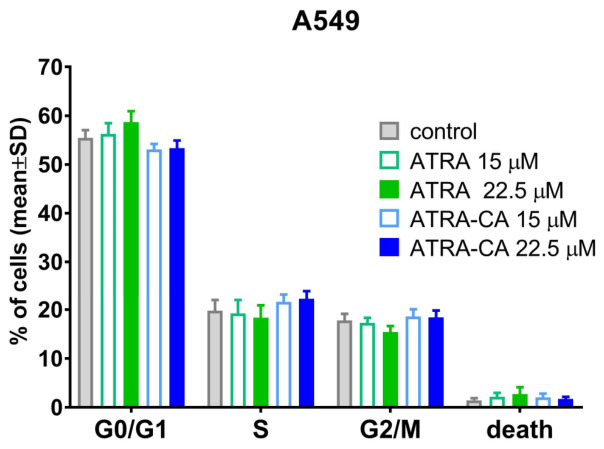
Cell cycle of lung cancer A549 cells after 72 h incubation with ATRA and ATRA-CA.

Lung cancer A549 cells were incubated for 72 h with two different (IC_50_: 15 μM and 1.5 × IC_50_: 22.5 μM) concentrations of ATRA and ATRA-CA and cell cycle phases were studied by using flow cytometry. We did not observe a significant influence of ATRA and ATRA-CA in the used concentrations on cell cycle (Figure 2). It can be observed that ATRA little increases number of death cells and cells in the G0/G1 phase and ATRA-CA little decreased number of cells at the G0/G1 phase but stopped cells in the S phase.

### 2.5. Cell Death Determination of Lung Cancer Cells After Incubation with ATRA and ATRA-CA

**Figure 3:**
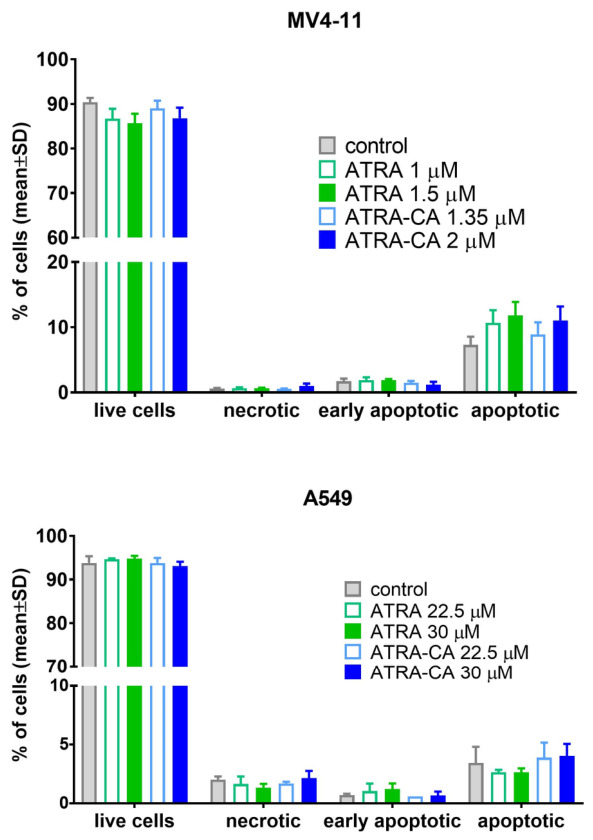
Cell death (apoptosis and necrosis) of leukemia MV4-11 and lung cancer A549 cells after 72 h incubation with ATRA and ATRA-CA.

The cells were exposed for 72 h to the compounds at concentrations as follows: leukemia MV4-11 cells: 1 and 1.5 µM for ATRA and 1.35 and 2 µM for ATRA-CA (about 1 × IC_50_ and 1.5 × IC_50_ value) and lung cancer A549 cells: 22.5 and 30 µM (about 1.5 × IC_50_ and 2 × IC_50_ value). Then, the cells were stained with Annexin V (AnV) and propidium iodide (PI) and cell death was determined using flow cytometry. The cells AnV−/PI− were live cells, AnV+/PI− early apoptotic, AnV+/PI+ apoptotic and AnV−/PI+ necrotic. No significance induction of cell death (apoptotic or necrotic) was observed by ATRA or ATRA-CA in used concentrations, only a small increase in apoptotic leukemia cells was observed (Figure 3).

### 2.6. Antiproliferative Activity of ATRA and ATRA-CA in Combination with Cytostatics

Combination therapy, which is a treatment that combines two or more therapeutic agents, enhances efficacy compared to the monotherapy approach because it targets key pathways in a characteristically synergistic or an additive manner. This approach potentially reduces drug resistance, while simultaneously providing therapeutic anticancer benefits, such as reducing tumor growth and metastatic potential, reducing cancer stem cell populations, and inducing apoptosis. As a result, combinations of anticancer agents could reduce toxicity and increase the efficacy of therapy.

Several studies were conducted on the anticancer effects of the combination treatment of all-*trans*-ATRA with other compounds and have shown stronger anticancer activities in vitro and in vivo. The combination treatment of all-*trans*-ATRA with, e.g., trastuzumab or epirubicin (against breast cancer cells), paclitaxel (against melanoma, hepatocellular carcinoma cells), gefitinib (against lung cancer cells), and gemcitabine (against pancreatic cancer cells) inhibited cell proliferation, invasion, and migration stronger than agents alone. Suppression of tumor development in an in vivo xenograft study were observed in the treatment of ATRA with vorinostat (melanoma) or paclitaxel (hepatocellular carcinoma) [48].

The anticancer activity of ATRA and ATRA-CA in combination with three different cytostatic drugs used in clinical practice—doxorubicin, cisplatin and paclitaxel—were examined. The lung A549 cancer cells and breast MCF-7 cancer cells were pre-treated for 1 h with ATRA and ATRA-CA in two concentration, 5 and 10 µM, and then cytostatics were added in four different concentrations for the next 72 h. The IC_50_ values for cytostatic alone, cytostatic with ATRA or ATRA-CA combination and ATRA and ATRA-CA alone, were calculated. Based on IC_50_ values for all compounds alone and for cytostatics in combination with retinoids, the combination index (CI) was calculated using the Chou and Talalay method [49].

#### 2.6.1. Combination with Cisplatin

**Figure 4:**
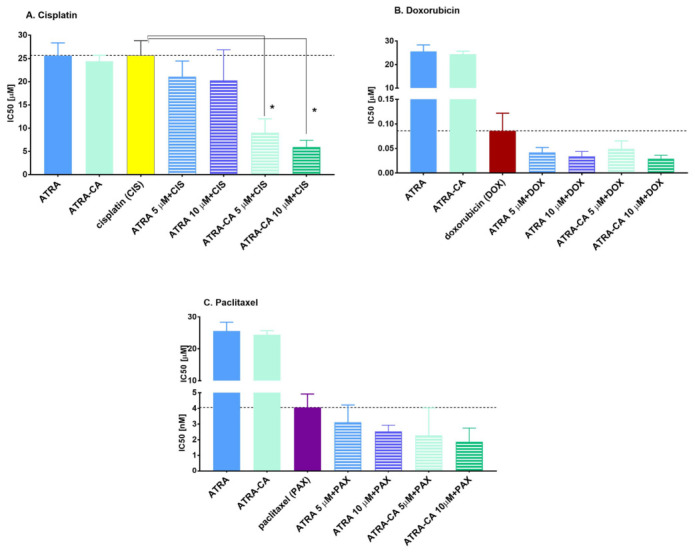
Antiproliferative activity of combined treatment of ATRA and ATRA-CA with cytostatic against human lung cancer A549 cells. The mean IC_50_ values for combination of ATRA or ATRA-CA (in concentration of 5 and 10 µM) with cisplatin (**A**), doxorubicin (**B**) and paclitaxel (**C**). * *p* < 0.05 for combined treatment in comparison to cytostatic alone, Kruskal–Wallis test, GraphPad Prism 7.

The treatment of A549 cells with ATRA together with cisplatin had slight influence on cisplatin activity. The IC_50_ value for cisplatin alone was 25.4 ± 3 µM, and with 5 or 10 µM of ATRA was 21.1 ± 3.4 and 20.3 ± 6.6 µM, respectively (Figure 4A). Calculation of combination index showed an additive effect (CI = 1.18) and antagonism (CI = 1.66) for these two combinations (Table 6). However, ATRA-CA had excellent influence on the anticancer activity of cisplatin. ATRA-CA in concentrations of 5 µM and 10 µM statistically significantly (*p* < 0.05) decreased IC_50_ for cisplatin from 25.4 ± 3 µM to 9 ± 3 µM and 5.9 ± 1.5 µM, respectively (Figure 4A), and we observed a synergistic (CI = 0.63) or additive (CI = 0.88) effect (Table 6).

**Figure 5:**
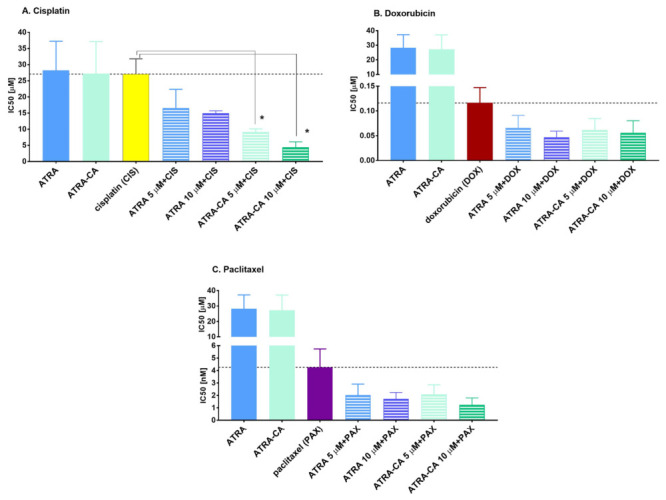
Antiproliferative activity of combined treatment of ATRA and ATRA-CA with cytostatic against human breast cancer MCF-7 cells. The mean IC_50_ values for combination of ATRA or ATRA-CA (in concentrations of 5 and 10 µM) with cisplatin (**A**), doxorubicin (**B**) and paclitaxel (**C**). * *p* < 0.05 for combined treatment in comparison to cytostatic alone, Kruskal–Wallis test, GraphPad Prism 7.

A similar effect was observed against breast cancer MCF-7 cells. ATRA decreased IC_50_ value for cisplatin alone from 26.6 ± 4.1 µM to 16.6 ± 5.8 µM (CI = 0.9 at 5 µM) and 14.9 ± 0.8 µM (CI = 1.19 at 10 µM), giving an additive effect. A high synergistic effect was observed for ATRA-CA and cisplatin. ATRA-CA in concentrations 5 or 10 µM diminished (statistically significant) IC_50_ for cisplatin to 9.1 ± 0.9 µM with CI = 0.58, and to 4.4 ± 1.7 µM with CI = 0.61, respectively (Figure 5A and Table 6).

#### 2.6.2. Combination with Doxorubicin

The treatment of A549 and MCF-7 cells with ATRA or ATRA-CA together with doxorubicin (DOX) lowered 1.8–2.5 times IC_50_ for cytostatic and the additive effect was observed (Figure 4B and Figure 5B, and Table 6). The IC_50_ value for doxorubicin alone was 0.081 ± 0.03 µM against A549 cells and was decreased in combination with 5 µM of ATRA or ATRA-CA to values 0.04 ± 0.01 µM and 0.05 ± 0.016 µM, respectively. In the combinations of DOX with 10 µM of ATRA or ATRA-CA the IC_50_ values were 0.03 ± 0.01 µM and 0.029 ± 0.007 µM, respectively. No significance differences in inhibition of cell growth were observed between the combinations of doxorubicin with ATRA and doxorubicin with ATRA-CA. The retinoids in a lower concentration of 5 µM had a slightly better influence on doxorubicin activity with lower values of CI in comparison to CI values for its combination with 10 µM of retinoids (Table 6).

In breast cancer MCF-7 cells, the IC_50_ values for DOX alone were 0.118 ± 0.034 µM and were diminished by adding of 5 µM of ATRA or ATRA-CA to values 0.066 ± 0.025 µM and 0.062 ± 0.023 µM, respectively. The addition of a higher concentration of ATRA or ATRA-CA (10 µM) decreased IC_50_ for DOX to the values 0.047 ± 0.013 µM and 0.056 ± 0.024 µM, respectively. The retinoids had a similar influence on doxorubicin activity and in combination with a lower (5 µM) concentration of the ATRA or ATRA-CA, the CI values were slightly lower (0.85 and 0.82, respectively) in comparison to CI values for its combination with 10 µM of retinoids (0.95 and 1.03, respectively).

#### 2.6.3. Combination with Paclitaxel

The treatment of A549 cells with 5 µM ATRA or ATRA-CA together with paclitaxel (PAX) had a slight influence on the cytostatic activity and the additive effect (CI 1.11 and 1.1) was observed (Table 6). The IC_50_ value for PAX alone was 4.03 ± 0.61 nM and with ATRA or ATRA-CA was 3.11 ± 1.11 and 3.02 ± 1.15 nM, respectively (Figure 4C). The addition of retinoids in a higher concentration of 10 µM decreased IC_50_ values for PAX to 2.54 ± 0.39 and 1.87 ± 0.87 nM, respectively (Figure 4C), but the calculated combination index (1.4 and 1.23) indicated the antagonism of this combination (Table 6).

The combination therapy using breast cancer MCF-7 cells showed much better effects (Table 6 and Figure 5C). The synergism in the combination of PAX with 5 µM of ATRA or ATRA-CA was observed and the IC_50_ values for paclitaxel alone, 3.96 ± 1.14 nM, was diminished two times to values 2.02 ± 0.89 nM (CI = 0.73) and 2.08 ± 0.78 nM (CI = 0.76), respectively. The higher concentration of retinoids (10 µM) increased the activity of combined treatment, reducing the IC_50_ for PAX to values 1.74 ± 0.50 nM and 1.25 ± 0.54 nM, respectively, and the calculated CI values 1.06 and 0.85 indicated additive effects. The combination treatments of paclitaxel and ATRA had similar anticancer effects like the combination with ATRA-CA, causing a decrease in IC_50_ for paclitaxel and the synergistic or additive effects.

#### 2.6.4. Determination of Influence of ATRA and ATRA-CA in Combination with Cytostatic on Cell Death

**Figure 6:**
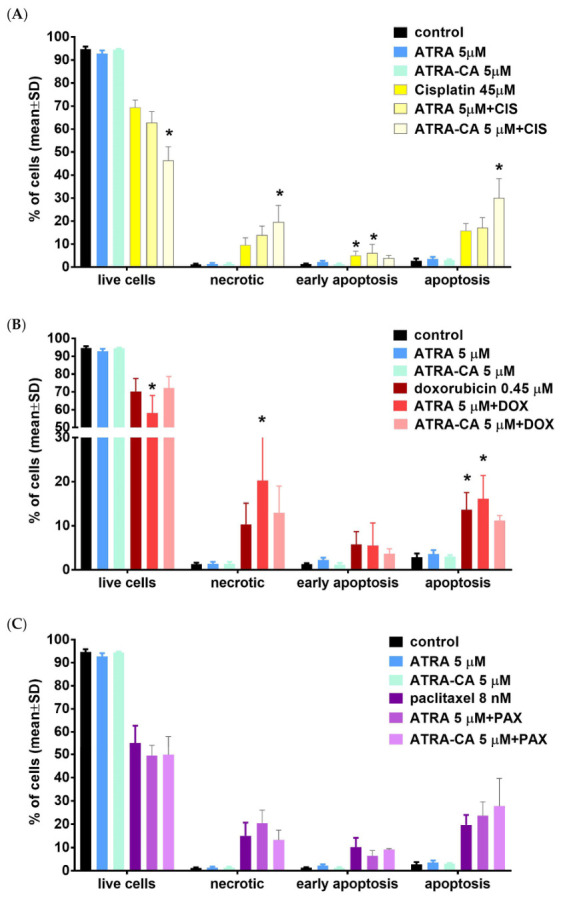
Cell death analysis of human lung cancer A549 cells after combined treatment of ATRA and ATRA-CA with cytostatics: cisplatin (**A**), doxorubicin (**B**) and paclitaxel (**C**). * *p* < 0.05 for combined treatment in comparison to cytostatic alone, Kruskal–Wallis test, GraphPad Prism 7.

**Figure 7:**
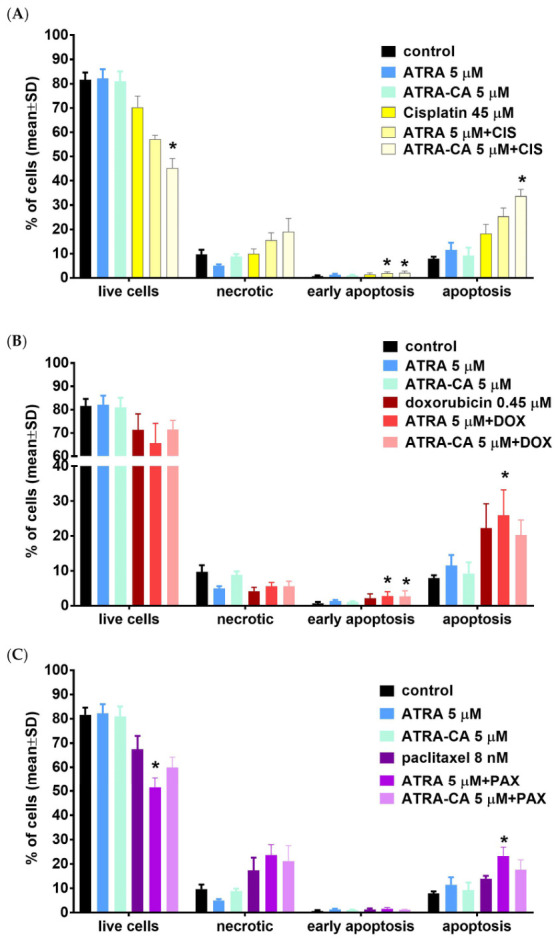
Cell death analysis of human breast cancer MCF-7 cells after combined treatment of ATRA and ATRA-CA with cytostatics: cisplatin (**A**), doxorubicin (**B**) and paclitaxel (**C**). * *p* < 0.05 for combined treatment in comparison to cytostatic alone, Kruskal–Wallis test, GraphPad Prism 7.

The induction of cell death (apoptosis or necrosis) of combined treatments 5 µM ATRA or ATRA-CA with cisplatin (45 µM), doxorubicin (0.45 µM) or paclitaxel (8 nM) were studied after 72 h incubation of lung cancer A549 and breast cancer MCF-7 cells. The apoptosis and necrosis of cells were determined by using Annexin V and DAPI staining. ATRA-CA with cisplatin statistically significantly (*p* < 0.05) induced apoptosis and necrosis of A549 and MCF-7 cells, stronger than cisplatin alone (Figure 6 and Figure 7).

ATRA with doxorubicin induced cell death (apoptosis and necrosis) slightly stronger than doxorubicin alone or doxorubicin with ATRA-CA. Also, combination of ATRA or ATRA-CA with paclitaxel had higher influence on induction of cell death than paclitaxel alone.

## 3. Materials and Methods

### 3.1. Substrates

The substrate all-*trans*-retinoic acid (ATRA) was purchased from BOC Sciences (Shirley, NY, USA) whereas cetyl alcohol (CA-ol), stearyl alcohol (SA-ol) and oleyl alcohol (OA-ol), as well as reagents 4-(*N*,*N*-dimethylamino)pyridine (DMAP) and *N*,*N*′-dicyclohexylcarbodiimide (DCC) were purchased from Sigma-Aldrich (St. Louis, MO, USA). Organic solvents (reagent grade) were used without further purification and were purchased from Sigma-Aldrich (St. Louis, MO, USA) and Chempur (Piekary Śląskie, Poland). Cytostatics used in combined therapy, cisplatin (Cisplatin 1 mg/mL Concentrate for Solution for Infusion), doxorubicin (Doxorubicinum 2 mg/mL) and paclitaxel (Paclitaxelum, 6 mg/mL), were purchased from Accord (Warsaw, Poland).

### 3.2. Methods of Analysis

Reactions were monitored by TLC, run on silica gel-coated aluminum sheets (silica gel 60 F_254_, Merck Ltd., Darmstadt, Germany). Chromatograms were visualized by spraying the plates with a solution of 1% Ce(SO_4_)_2_ and 2% H_3_[P(Mo_3_O_10_)_4_] in 10% H_2_SO_4_ and heating the plates to 200 °C. Purification was performed using column chromatography with silica gel 60 (230–400 mesh ASTM, Merck, Darmstadt, Germany).

The chemical structure of synthesized compounds was confirmed by NMR spectroscopy. All of the ^1^H, ^13^C NMR spectra were recorded using Brucker Advance II 600 MHz spectrometer (Brucker, Rheinstetten, Germany). Samples were dissolved in CDCl_3_/CD_3_OD 2:1 (*v*/*v*). HRMS spectra were obtained for all samples of the synthesized compounds using an electron spray ionization (ESI) technique on a Waters ESI-Q-TOF Premier XE spectrometer (Waters Corporation, Milford, MA, USA).

### 3.3. Synthesis of Ester Derivatives of All-Trans-Retinoic Acid

Retinoic acid (ATRA) (200 mg, 0.66 mmol), DMAP (122 mg, 1 mmol) and fatty alcohols (CA-ol or SA-ol or OA-ol) (1.32 mmol) were dissolved in CH_2_Cl_2_ (8 mL), and DCC (206 mg, 1 mmol) was added. The mixture was stirred for 24 h at room temperature in a dark vial without access to light. After completion of the reaction, the product was extracted with hexane. The organic solvent was then evaporated, and the resulting crude product was purified by column chromatography on silica gel using methylene chloride/hexane (2:3, *v*/*v*) as the eluent. Physical and spectroscopic data of pure products are given below:

#### 3.3.1. Cetyl-All-*Trans*-Retinate (ATRA-CA)

(purity 97%) was previously reported in the literature, and the obtained spectroscopic data are consistent with those described in Ref. [27].

#### 3.3.2. Stearyl-All-*Trans*-Retinate (ATRA-SA)

Yellow solid (75% yield, R_f_ = 0.34, purity 95%); ^1^H NMR (600 MHz, CDCl_3_/CD_3_OD 2:1 (*v*/*v*), δ: 0.79 (t, *J* = 7.0 Hz, 3H, CH_3_-18′), 0.95 (s, 6H, CH_3_-16 and CH_3_-17), 1.18 (m, 30H, CH_3_(CH_2_)_15_CH_2_CH_2_OC(O)-, 1.39 (m, 2H, CH_2_-2), 1.52–1.60 (2m, 4H, CH_2_-3, CH_2_-2′), 1.63 (s, CH_3_-18), 1.93 (s, CH_3_-19), 1.95 (m, 2H CH_2_-4), 2.26 (s, CH_3_-20), 4.02 (t, *J* = 6.6 Hz, 2H, CH_2_-1′), 5.70 (s, 1H, H-14), 6.05–6.23 (2m, H-7, H-8, H-11, H-12), 6.94 (dd, *J* = 15.1 and 3.6 Hz, 1H, H-10); ^13^C NMR (150 MHz, CDCl_3_/CD_3_OD 2:1 (*v*/*v*), δ: 12.60 (C-19), 13.63 and 13.80 (C-18′, C-20), 19.10 (C-3), 21.44 (C-18), 22.55, 25.91, 28.71, 29.14, 29.25, 29.39, 29.43, 29.51, 29.54, 32.96, 34.13 (C-3′-C-17′), 28.60 (C-2′), 29.57 (C-16 and C-17), 31.82 (C-4), 39.52 (C-2), 64.07 (C-1′), 118.27 (C-14), 128.67, 129.39 (C-11, C-12), 129.87 (C-6), 131.14 (C-10), 134.96, 137.25 (C-7, C-8), 139.67 (C-1 and C-9), 152.98 (C-13), 167.87 (C-15). ESI-MS *m*/*z* calculated for C_38_H_64_O_2_: [M+H]^+^ 553.4985. Found 553.4998.

#### 3.3.3. Oleyl-All-*Trans*-Retinate (ATRA-OA)

Yellow solid (58% yield, R_f_ = 0.29, purity 96%); ^1^H NMR (600 MHz, CDCl_3_/CD_3_OD 2:1 (*v*/*v*), δ: 0.80 (t, *J* = 7.1 Hz, 3H, CH_3_-18′), 0.95 (s, 6H, CH_3_-16 and CH_3_-17), 1.18–1.30 (m, 24H, CH_3_(CH_2_)7C==C(CH_2_)_7_ CH_2_OC(O)-, 1.39 (m, 2H, CH_2_-2), 1.53–1.59 (2m, 4H, CH_2_-3, CH_2_-11′), 1.63 (s, CH_3_-18), 1.89 (m, 2H, CH_2_-4), 1.93 (s, CH_3_-19), 1.94 (m, 2H, CH_2_-8′), 2.26 (s, CH_3_-20), 4.02 (t, *J* = 6.6 Hz, 2H, CH_2_-1′), 5.25–5.27 (2m, 2H, H-9′ and H-10′), 5.70 (s, 1H, H-14), 6.05–6.23 (2m, 4H, H-7, H-8, H-11, H-12), 6.94 (dd, *J* = 15.0 and 3.5 Hz, 1H, H-10); ^13^C NMR (150 MHz, CDCl_3_/CD_3_OD 2:1 (*v*/*v*), δ: 12.60 (C-19), 13.63 and 13.80 (C-18′ and C-20), 19.10 (C-3), 21.45 (C-18), 22.55, 25.91, 27.04, 29.05, 29.13, 29.19, 29.21, 29.28, 29.40, 29.60, 29.65, 34.13 (C-2′-C-7′, C-12′-C-17′), 28.60 (C-11′), 28.72 (C-16 and C-17), 28.80, 29.10, 29.17, 29.23, 29.32, 29.44, 29.64, 29.68, 31.83 (C-2′-C-17′), 31.81 (C-4), 32.96 (C-8′), 39.52 (C-2); 64.05 (C-1′), 118.26 (C-14), 128.67 and 129.40 (C-11 and C-12), 129.70 and 129.85 (C-9′ and C-10′), 131.15 (C-10), 134.96 and 137.25 (C-7 and C-8), 137.65 and 139.65 (C-1, C-9, C-5, C-6), 152.99 (C-13), 167.85 (C-15). ESI-MS *m*/*z* calculated for C_38_H_62_O_2_: [M+H]^+^ 551.4828. Found 551.4818.

### 3.4. Hydrolytic Stability of Synthesized Cetyl-All-Trans-Retinate (ATRA-CA)

The stability of synthesized cetyl-all-*trans*-retinate (ATRA-CA) at pH 1.2 and 7.2 was assessed using Clark-Lubs and PBS buffers, respectively [50]. The ester was dissolved at a concentration of 4 mg/mL (n = 3) and incubated under agitation (300 rpm) at 37 °C. For stability assessment, samples were collected at predetermined time points: 0.5, 1, 2, 4, 8, 12, and 24 h. At each time point, 100 µL aliquots were withdrawn, evaporated to dryness under a nitrogen atmosphere, and reconstituted in 400 µL of methanol (MeOH) for further analysis. Hydrolysis was qualitatively analyzed using thin-layer chromatography (TLC). Samples were also analyzed by high-performance liquid chromatography (HPLC) using a Shimadzu LC-2050C 3D system (Shimadzu, Kyoto, Japan) equipped with an evaporative light scattering detector (ELSD). The autosampler and column temperatures were maintained at 30 °C. Separation was performed on a YMC Carotenoid column (5 µm particle size; YMC Co., Kyoto, Japan). The injection volume was 10 µL, and the flow rate was set at 0.8 mL/min under gradient elution for a total run time of 24 min. The mobile phases consisted of solvent A (water), solvent B (methanol), and solvent C (methyl tert-butyl ether, MTBE). The gradient program was as follows: 0–8 min, 10/90/0 (%A/%B/%C, *v*/*v*/*v*); 10–18 min, 0/35/65; 20–24 min, 10/90/0.

### 3.5. Drug Nature and in Silico Pharmacokinetics and Toxicological Profile

The prediction of physicochemical properties of retinoic acid esters was based on their molecular structures. The topological polar surface area (TPSA), octanol-water partition coefficient (logP), and the number of hydrogen bond acceptors and donors were calculated using the Molinspiration online [51] (https://www.molinspiration.com/) (accessed on 1 March 2026) property calculation toolkit. In contrast, the molecular descriptors related to geometry, such as surface area, volume, and ovality, were determined using the SPARTAN’18 software (Wavefunction, Inc., Irvine, CA, USA), which enables advanced quantum mechanical calculations. The potential biological activity of the studied esters was assessed using the Way2Drug PASS Online service (Prediction of Activity Spectra for Substances; http://www.way2drug.com/PASSonline/) (accessed on 1 March 2026). This system predicts biological activity in terms of two independent probabilities: the probability of a given activity being exhibited (Pa) and the probability of it not being exhibited (Pi). Both values range from 0 to 1, where a higher Pa value indicates a greater likelihood of a specific biological activity occurring. Pharmacokinetic parameters, including absorption, distribution, metabolism, and excretion (ADME), as well as toxicity, were predicted using pkCSM (http://biosig.unimelb.edu.au/pkcsm/) (accessed on 1 March 2026) [52,53].

### 3.6. Biological Studies

#### 3.6.1. Cell Lines and Cultured Mediums

All cell lines used in the research were stored in a cell bank at the Hirszfeld Institute of Immunology and Experimental Therapy, PAS, Wroclaw, Poland. Human cell lines: leukemia MV4-11, gastric cancer AGS, colon cancer HT-29 and normal breast MCF-10A were obtained from ATCC (Manassas, VA, USA), lung cancer A549 and breast cancer MCF-7 were obtained from ECACC (Salisbury, UK) and breast cancer MDA-MB-468 were obtained from DSMZ (Braunschweig, Germany).

The cell lines MV4-11 and MDA-MB-468 were cultured in RPMI 1640 medium (IIET PAS, Wrocław, Poland) with the addition of 1.0 mM sodium pyruvate (only MV4-11) and 10% (MV4-11) or 20% (MDA-MB-468) fetal bovine serum (FBS) (all from Merck, Darmstadt, Germany). The A549, HT-29 and AGS cells were cultured in RPMI 1640 + Opti-MEM (1:1) (IIET PAS, Wrocław, Poland and Gibco, Paisley, UK) with the addition of 5% FBS (Merck, Germany) and 1.0 mM sodium pyruvate (only HT-29). The MCF-7 cells were cultured in Eagle medium (IIET PAS, Wrocław, Poland) with the addition of 10% FBS, 8 microg/mL of insulin and 1% of MEM NON-Essential amino acid (all Merck, Darmstadt, Germany). Normal breast epithelial MCF-10A cells were cultured in the HAM’S F-12 medium (Corning Life Sciences, Tewksbury, MA, USA), which was supplemented with 10% Horse Serum (Gibco, Dreieich, Germany), 20 ng/mL EGFh, 10 µg/mL insulin, 0.5 µg/mL Hydrocortisone and 0.05 mg/mL Cholera Toxin from Vibrio cholerae (all from Merck, Darmstadt, Germany). All culture media were supplemented with 2 mM L-glutamine (Merck, Germany), 100 units/mL penicillin, (Polfa Tarchomin S.A., Warsaw, Poland) and 100 µg/mL streptomycin (Merck, Darmstadt, Germany). All cell lines were grown at 37 °C with 5% CO_2_ humidified atmosphere.

#### 3.6.2. Determination of Antiproliferative Activity

The antiproliferative activity of ATRA and its esters were tested against six cancer cell lines (MV4-11, A549, HT-29, AGS, MDA-MB-468, MCF-7). Their cytotoxicity was also tested against one normal breast cell line MCF-10A. The tested compounds were diluted in DMSO or EtOH (96%) to the concentration of 50 mM and then in culture medium to reach the final concentrations in the study. Before adding of the tested compounds (24 h prior), the cells were plated in 384-well plates (Greiner Bio-One, Kremsmünster, Austria) at a density of 1 × 10^3^ (A549) or 1.5 × 10^3^ (MDA-MB-468, HT-29, MCF-7) or 2 × 10^3^ (AGS, MCF-10A) cells per well. The MV4-11 cells were plated in 96-well plates (Corning) at a density of 5 × 10^3^ cells per well. The assay was performed after 72 h of exposure to 0.2, 0.63, 2.0, 6.33, 20, 63.3, 200 and 633 μM concentration of the retinoid’s solutions. The in vitro cytotoxic effect of all agents was examined using the MTT (MV4-11) or SRB assay (A549, HT-29, AGS, MDA-MB-468, MCF-7, MCF-10A) as described previously [54,55]. The results were calculated as an IC_50_ (inhibitory concentration 50%) of the concentration of tested agent, which is cytotoxic for 50% of the cancer cells. IC_50_ values were calculated for each experiment separately using Prolab-3 system based on Cheburator 0.4 software [56] and mean values ± SD are presented in Table 4. Each compound in each concentration was tested in triplicate in a single experiment, which was repeated 3–5 times.

#### 3.6.3. Determination of Antiproliferative Activity of ATRA and ATRA-CA in Combination with Cytostatics

The antiproliferative activity of ATRA and ATRA-CA in combination with cytostatics doxorubicin, cisplatin and paclitaxel, were tested against cancer cell lines A549 and MCF-7. Before adding of the tested compounds (24 h prior), the cells were plated in 96-well plates (Greiner Bio-One) at a density of 5 × 10^3^ (A549) or 7.5 × 10^3^ (MCF-7) cells per well. The assay was performed after 72 h of exposure of cells to ATRA and ATRA-CA alone or in combination with cytostatics (at concentrations of 5 or 10 μM). Then, after 1 h (the pre-treatment with retinoids) the cytostatics were added (in combination with retinoids and alone as monotherapy): cisplatin in concentrations 0.1–100 μM, doxorubicin in concentrations 0.01–10 μM, and paclitaxel in concentrations 0.1–100 nM. The in vitro cytotoxic effect (after 72 h exposure) was examined using the SRB assay as described previously [51,52]. The results were calculated as an IC_50_ (values were calculated for each experiment separately using the Prolab-3 system based on the Cheburator 0.4 software [53]). Experiments were repeated 3–5 times.

The cytotoxic effects obtained from the combinations of retinoids with the cytostatics drugs were analyzed according to the method of Chou and Talalay [49]. The interaction between two agents (mutually non-exclusive) was assessed by the means of combination index (CI) calculated for the mean IC_50_ from antiproliferative experiments (which were repeated 3–5 times):

CI_A+B_ = (D_A/A+B_/D_A_) + (D_B/A+B_/D_B_) + (D_A/A+B_ × D_B/A+B_)/D_A_ D_B_

where

CI_A+B_ is the combination index for the experimentally achieved effect F (IC_50_) for the combination of compound A (the given cytostatic) and compound B (retinoid ATRA or ATRA-CA),

D_A/A+B_—the concentration of compound A in the combination A + B giving the effect F,

D_B/A+B_—the concentration of compound B in the combination A + B giving the effect F,

D_A_—the concentration of compound A alone giving the effect F

D_B_—the concentration of compound B alone giving the effect F

A combination index of CI < 0.8 indicates synergism, CI > 1.2 antagonism, and CI = 0.8–1.2 indicates an additive effect. The IC_50_ mean values ± SD and CI are presented in Table 5, Figure 4 and Figure 5.

#### 3.6.4. Cell Cycle Analysis of Lung Cancer Cells After Incubation with ATRA and ATRA-CA

The A549 cells were seeded at a density of 0.7 × 10^5^ cells/well on 6-well plates (Greiner Bio-One) in culture medium to the final volume of 4 mL. The cells were exposed to the ATRA and ATRA-CA compounds at concentrations of 15 and 22.5 μM (concentrations approximately the same value of IC_50_ and 1.5 × IC_50_) for 72 h. After incubation, the cells were collected, and 1 × 10^6^ of cells were washed twice in cold PBS and fixed for 24 h in 70% ethanol at −20 °C. Then, the cells were washed twice in PBS and incubated with RNAse (8 μg/mL, Thermo Scientific, Waltham, MA, USA) at 37 °C for 1 h. The cells were stained for 30 min with propidium iodide (50 μg/mL, Merck, Germany) at 4 °C, and the cellular DNA content was analyzed by flow cytometry using BD LSRFortessa cytometer (BD Bioscience, USA). Compounds at each concentration were tested at least three times independently. Obtained results were analyzed using Flowing software 2.5.1 (Turku, Finland).

#### 3.6.5. Cell Death Determination by Annexin V and PI or DAPI Staining

##### Monotherapy: After Incubation with ATRA and ATRA-CA

The cells were seeded at the density of 0.75 × 10^5^ (MV4-11) or 0.45 × 10^5^ (A549) cells/well on 12-well plates (Greiner Bio-One) in culture medium to the final volume of 3 mL. The MV4-11 cells were exposed for 72 h to the compounds at concentrations 1 and 1.5 µM for ATRA and 1.35 and 2 µM for ATRA-CA (about 1 × IC_50_ and 1.5 × IC_50_ value). The A549 cells were exposed for 72 h to the compounds at concentrations 22.5 and 30 µM (about 1.5 × IC_50_ and 2 × IC_50_ value).

##### Combination Therapy: After Incubation with ATRA or ATRA-CA and with Cytostatics

The lung cancer A549 and breast cancer MCF-7 were seeded at the density of 0.45 × 10^5^ (A549) or 0.9 × 10^5^ (MCF-7) cells/well on 12-well plates (Greiner Bio-One) in culture medium to the final volume of 3 mL. The cells were exposed to the ATRA or ATRA-CA at concentrations of 5 μM and then (after 1 h pre-treatment) cells were exposed to the cytostatics for 72 h: cisplatin at concentrations of 45 μM, doxorubicin at concentrations of 0.45 μM or paclitaxel at concentrations of 8 nM.

After incubation in mono- or combination therapy, the cells were collected, counted, and 1 × 10^5^ of cells were washed twice with PBS and then suspended in 100 µL of binding buffer (Hepes buffer: 10 mM HEPES/NaOH, pH 7.4, 150 mM NaCl, 5 mM KCl, 1 mM MgCl_2_, 1.8 mM CaCl_2,_ (IIET, Wrocław, Poland) with 4 µL of APC-Annexin V (BD Pharmingen, Franklin Lakes, NJ, USA)). After 15 min of incubation in the dark at room temperature, prior to the analysis, the propidium iodide (PI) solution (final concentration of 4 µg/mL) or 4′,6-diamidino-2-phenylindole (DAPI) solution (final concentration of 0.4 µg/mL) was added. DAPI was used in the experiment of combination therapy.

Data acquisition was performed by flow cytometry using BD LSRFortessa cytometer (BD Bioscience, San Jose, CA, USA). Compounds at each concentration and combinations were tested at least three times independently. Results were analyzed using Flowing software 2.5.1 (Turku, Finland). The data were displayed as a two-color dot plot with an APC-Annexin V (AnV) vs. PI or DAPI. Double-negative cells were live cells, PI+/AnV+ or DAPI+/AnV+ were late apoptotic, PI−/AnV+ or DAPI−/AnV+ were early apoptotic cells and PI+/AnV− or DAPI+/AnV− were necrotic cells.

### 3.7. Statistical Analysis

The Kruskal–Wallis tests were performed for two-group comparisons to indicate the significant differences between treatments using GraphPad Prism 7. A statistically significant difference was considered at *p* ≤ 0.05.

## 4. Conclusions

This study provides proof-of-concept evidence that the synthesis of all-*trans*-retinoic acid (ATRA) prodrugs in the form of esters enables modulation of its anticancer activity and pharmacological profile. All synthesized esters demonstrated measurable antiproliferative activity against selected cancer cell lines while maintaining a favorable safety profile toward non-tumorigenic breast epithelial cells (MCF-10A). Among the evaluated compounds, ATRA-CA exhibited the greatest therapeutic potential.

Moreover, a nonlinear dependence of biological activity on alkyl chain length was observed, consistent with the literature-described cut-off effect, in which activity does not increase proportionally with increasing lipophilicity. ATRA-CA also enhanced the efficacy of standard chemotherapeutic agents, including cisplatin, doxorubicin, and paclitaxel, demonstrating synergistic or additive pharmacodynamic interactions and leading to reduced IC_50_ values.

Considering the results obtained in this study, future research should focus on the development of lipid-based delivery systems for cetyl all-*trans*-retinoate (ATRA-CA), as well as on mechanistic studies aimed at confirming its prodrug mode of action, including enzymatic hydrolysis assays, determination of ATRA release, and analysis of intracellular distribution.

## Figures and Tables

**Table 1:** Molecular descriptors of ATRA and ATRA-lipid derivatives calculated using SPARTAN’18 (volume, area, ovality) or Molinspiration (logP, TPSA, HBA, HBD) software.

Compound	LogP	TPSA (Å^2^)	Area (Å^2^)	Volume (Å^3^)	Ovality	HBA	HBD	Molecular Weight (g/mol)
ATRA	5.80	37.30	380.18	354.33	1.57	2	1	300.44
ATRA-CA	9.93	26.30	703.77	649.65	1.94	2	0	524.87
ATRA-SA	10.07	26.30	743.87	686.28	1.98	2	0	552.93
ATRA-OA	10.01	26.30	738.22	682.30	1.97	2	0	550.91

**Table 2:** Predicted activity of ATRA derivatives determined by the PASS method.

Activity	ATRA		ATRA-CA		ATRA-SA		ATRA-OA	
	Pa	Pi	Pa	Pi	Pa	Pi	Pa	Pi
All-*trans*-retinyl-palmitate hydrolase inhibitor	0.972	0.001	0.988	0.000	0.988	0.000	0.991	0.000
CYP2J substrate	0.988	0.001	0.977	0.001	0.977	0.001	0.982	0.001
CYP4A substrate	0.427	0.024	0.956	0.001	0.956	0.001	0.969	0.001
BRAF expression inhibitor	0.337	0.018	0.950	0.000	0.950	0.000	0.949	0.000
G-protein-coupled receptor kinase inhibitor	0.966	0.002	0.945	0.003	0.945	0.003	0.956	0.002
Antineoplastic	0.874	0.005	0.811	0.010	0.811	0.010	0.803	0.011
Apoptosis agonist	0.899	0.004	0.878	0.005	0.878	0.005	0.884	0.005
Anticarcinogenic	0.811	0.005	0.777	0.006	0.777	0.006	0.807	0.005
Chemoprotective	0.751	0.003	0.728	0.003	0.730	0.005	0.723	0.003
Chemopreventive	0.793	0.004	0.730	0.005	0.730	0.002	0.771	0.004

Pa—probable activity; Pi—probable inactivity. Values range from 0 to 1, where 1 represents 100% probability of Pa or Pi and 0 represents 0% probability of Pa or Pi.

**Table 3:** Predicted ADMET profiles of ATRA derivatives (pkCSM analysis).

Parameters	ATRA-CA	ATRA-SA	ATRA-OA
Absorption			
Caco-2 permeability (log Papp in 10^−6^ cm/s)	1.382	1.373	1.385
Intestine absorption (% Absorbed)	89.555	88.868	89.374
P-glycoprotein substrate	no	no	no
P-glycoprotein inhibitor I	no	no	no
P-glycoprotein inhibitor II	yes	yes	yes
Distribution			
VDss human (log L/kg)	−0.057	−0.19	−0.207
BBB permeability (log BB)	0.885	0.922	0.912
CNS permeability (log PS)	−1.299	−1.19	−1.136
Metabolism			
CYP2D6	yes	no	no
CYP3A4	yes	yes	yes
Excretion			
Total clearance (log mL/min/kg)	1.524	1.557	1.534
Renal OTC substrate	no	no	no
Toxicity			
AMES toxicity	no	no	no
Max tol. dose human (log mg/kg/day)	0.175	0.112	0.907
Oral rat acute toxicity (LD50 mol/kg)	1.872	1.937	1.632

**Table 4:** The half maximal inhibitory concentrations (IC_50_) of ATRA and its derivatives against selected cancer cell lines and non-tumorigenic human breast epithelial cell line (MCF-10A).

Compound	Cell Lines IC_50_ [µM]						
	MV4-11	A549	HT-29	AGS	MDA-MB-468	MCF-7	MCF-10A
ATRA	1.1 ± 0.1	14.1 ± 5.1	11.1 ± 5.2	21.8 ± 3.5	78.2 ± 15.1	22 ± 13.6	52.6 ± 20.4
CA-ol	53.3 ± 8.9	33.7 ± 1.2	30.6 ± 2.5	72.6 ± 15.8	112.2 ± 2	99.2 ± 4.5	107.9 ± 3.7
SA-ol	75.3 ± 17.3	109.2 ± 11.4	43.2 ± 0.3	178.1 ± 73.3	271.3 ± 133.2	129.5 ± 16.2	350.5 ± 32.2
OA-ol	62.2 ± 2.1	119.3 ± 34	80.2 ± 27.4	107.7 ± 4.5	345.1 ± 20.8	131.6 ± 36.3	307.8 ± 59.3
ATRA-CA	1.3 ± 0.7	12.9 ± 6.3	8.5 ± 1.2	8.78 ± 1.2	23.1 ± 1.8	8.3 ± 3	72.4 ± 39.1
ATRA-SA	112.7 ± 29.3	168.9 ± 79.3	364.5 ± 149.4	179.8 ± 101.7	496.8 ± 112.7	197 ± 91.7	396.2 ± 140.5
ATRA-OA	30.9 ± 10.0	65.9 ± 17.3	88.9 ± 18.6	93.6 ± 7.8	102.4 ± 5.8	91.4 ± 19	135.6 ± 27.1

Studied concentrations 0.2–633 µM. Data are presented as mean ± standard deviation (SD) calculated using Prolab-3 system based on Cheburator 0.4 software.

**Table 5:** The selectivity index (SI) of tested retinoids.

Compound	Cell Lines/Calculated Selectivity Index SI					
	MV4-11	A549	HT-29	AGS	MDA-MB-468	MCF-7
ATRA	48.7	3.72	4.76	2.41	0.67	2.39
CA-ol	2.02	3.2	3.53	1.49	0.96	1.09
SA-ol	4.65	3.21	8.11	1.97	1.29	2.71
OA-ol	4.95	2.58	3.84	2.86	0.89	2.34
ATRA-CA	54.03	5.56	8.52	8.25	3.13	8.68
ATRA-SA	3.51	2.35	1.09	2.2	0.8	2.01
ATRA-OA	4.38	2.06	1.53	1.45	1.32	1.48

The SI = IC_50_ for normal cell line (MCF-10A)/IC_50_ for respective cancerous cell line. A beneficial SI > 1.0 indicates a drug with efficacy against tumor cells greater than toxicity against normal cells.

**Table 6:** Combinations index (CI) values of combination therapy of ATRA or ATRA-CA with cytostatics.

Cytostatic	Index CI							
	A549				MCF-7			
	+ATRA		+ATRA-CA		+ATRA		+ATRA-CA	
	5 µM	10 µM	5 µM	10 µM	5 µM	10 µM	5 µM	10 µM
CIS	1.18	1.66	0.63	0.88	0.9	1.19	0.58	0.61
DOX	0.78	1.13	0.89	1.1	0.85	0.95	0.82	1.03
PAX	1.11	1.4	1.1	1.23	0.73	1.06	0.76	0.85

CI calculated according to the Chou and Talalay method. CI < 0.8: synergism (marked in green font); CI = 0.8–1.2: additive effect (marked in blue font); CI > 1.2: antagonism (marked in black font).

## Data Availability

All data generated or analyzed during this study are included in this published article and its Supplementary Information Files.

## Supplementary Materials

The following supporting information can be downloaded at https://www.mdpi.com/article/10.3390/ijms27135982/s1.

## Abbreviations

The following abbreviations are used in this manuscript:

A549	lung cancer
AGS	gastric cancer
AML	acute myeloid leukemia
APL	acute promyelocytic leukemia
ATO	arsenic trioxide
ATRA	all-*trans*-retinoic acid, tretinoin
ATRA-CA	cetyl-all-*trans*-retinate
ATRA-OA	oleyl-all-*trans*-retinate
ATRA-SA	stearyl-all-*trans*-retinate
BBB	blood–brain barrier
CA-ol	cetyl alcohol
CI	combination index
CIS	cisplatin
COSY, HSQC	correlation spectroscopy
DAPI	4′,6-diamidino-2-phenylindole
DCC	*N*,*N*′-dicyclohexylcarbodiimide
DMAP	4-(*N*,*N*-dimethylamino)pyridine
DOX	doxorubicin
ELSD	evaporative light scattering detector
ESI-MS	mass spectrometry
HBA	hydrogen bond acceptor
HBD	hydrogen bond donor
HT-29	colon cancer
IC50	half maximal inhibitory concentration
logP	octanol-water partition coefficient
MCF-7	hormone receptor-positive breast cancer
MDA-MB-468	triple-negative breast cancer
MTBE	methyl tert-butyl ether
MV4-11	leukemia
NMR	nuclear magnetic resonance
OA-ol	oleyl alcohol
OTC	organic cation transporter
Pa	probability of being active
PASS	Prediction of Activity Spectra for Substance
PAX	paclitaxel
Pi	probability of being inactive
PI	propidium iodide
RARs	retinoic acid receptors
RAS	retinoic acid syndrome
RXRs	retinoid X receptors
SA-ol	stearyl alcohol
SAR s	structure–activity relationship
SD	standard deviation
SI s	electivity index
TPSA	topological polar surface area
