# Supplementary material for: Cetyl All-Trans-Retinoate as a Lipidic ATRA Prodrug with Enhanced Anticancer and Chemosensitizing Activity
Source: Int J Mol Sci. 2026 Jul 3;27(13):5982. doi: 10.3390/ijms27135982 (PMC13361345; doi:10.3390/ijms27135982)
Supplement: Supplementary file 1 [file ijms-27-05982-s001.zip › ijms-4350473-supplementary.pdf]

Supplementary Materials

Cetyl all-Trans-Retinoate as a Lipidic ATRA Prodrug with Enhanced Anticancer and Chemosensitizing Activity

Paweł Moroz [1], Klaudia Muciek [1], Marta Świtalska [2], Joanna Wietrzyk [2], Zbigniew Lazar [3] and Anna Gliszczyńska [1,*]

[1] Department of Food Chemistry and Biocatalysis, Wrocław University of Environmental and Life Sciences, Norwida 25, 50-375 Wrocław, Poland
[2] Department of Experimental Oncology, Hirszfeld Institute of Immunology and Experimental Therapy, Polish Academy of Sciences, Weigla 12, 53-114 Wrocław, Poland
[3] Department of Biotechnology and Food Microbiology, Wrocław University of Environmental and Life Sciences, Chelmonskiego 37, 51-630 Wrocław, Poland
* Correspondence: anna.gliszczynska@upwr.edu.pl

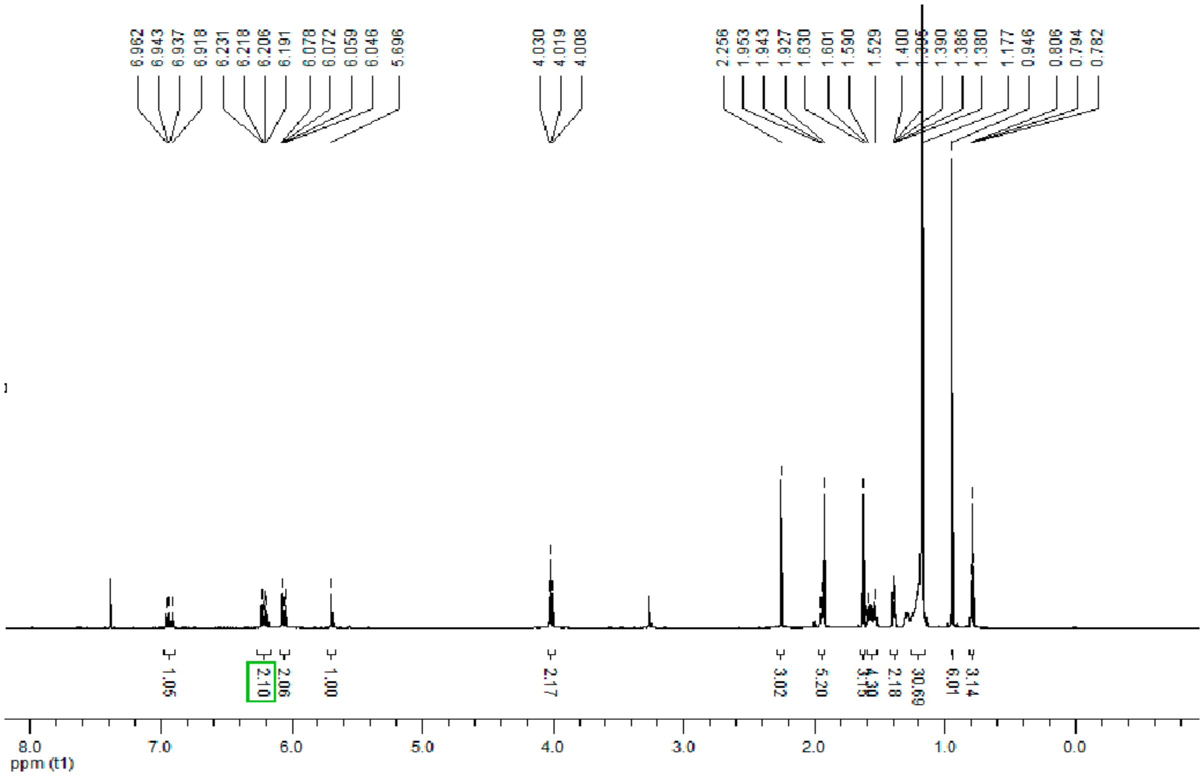

Figure S1. ^{1}H NMR spectrum (600 MHz, $CDCl_3/CD_3OD$) of ATRA-SA.

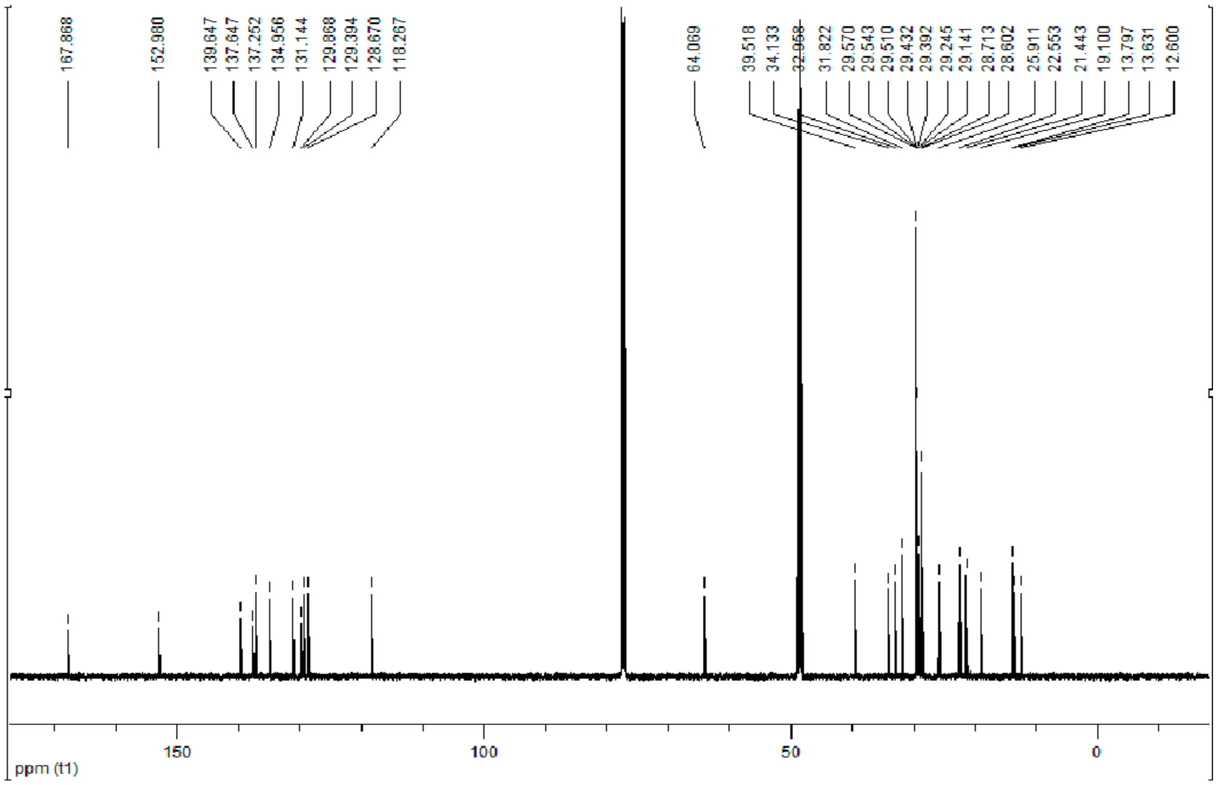

Figure S2. ^{13}C NMR spectrum (150 MHz, $CDCl_3/CD_3OD$) of ATRA-SA.

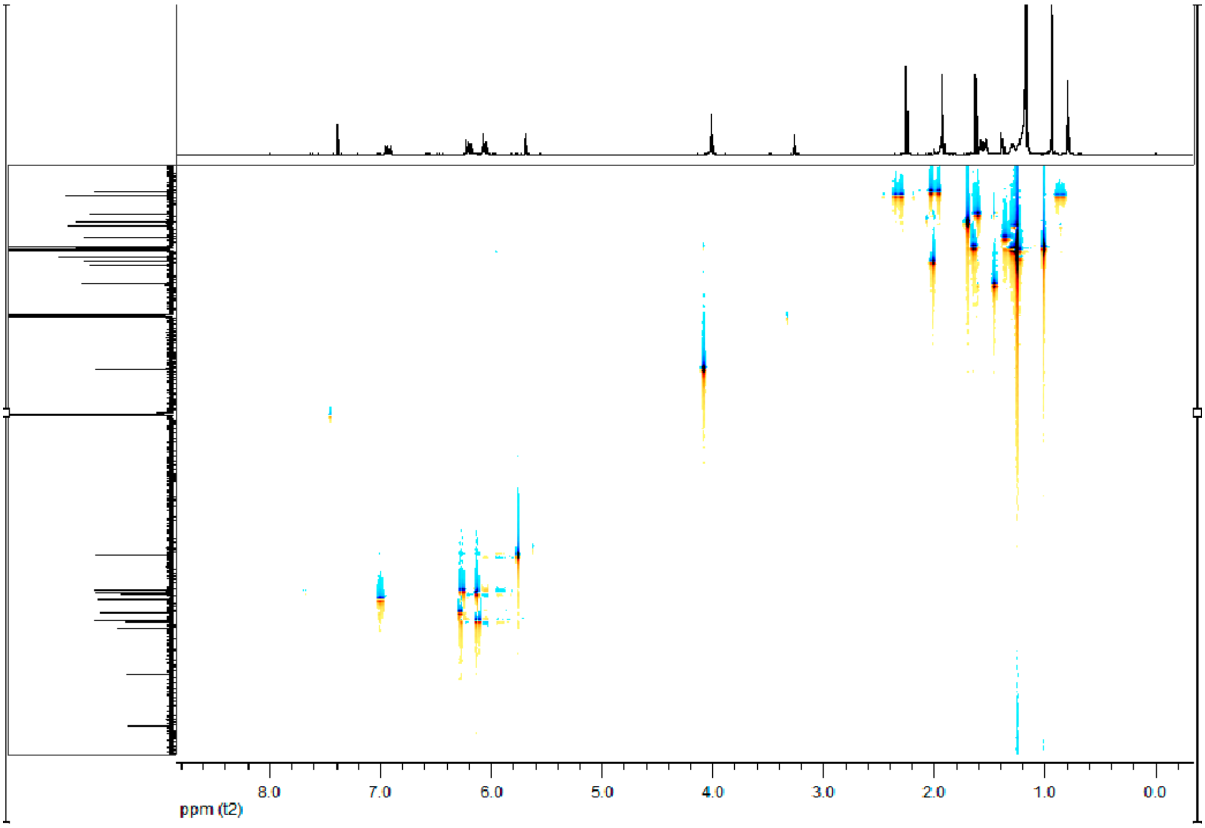

Figure S3. COSY spectrum of ATRA-SA.

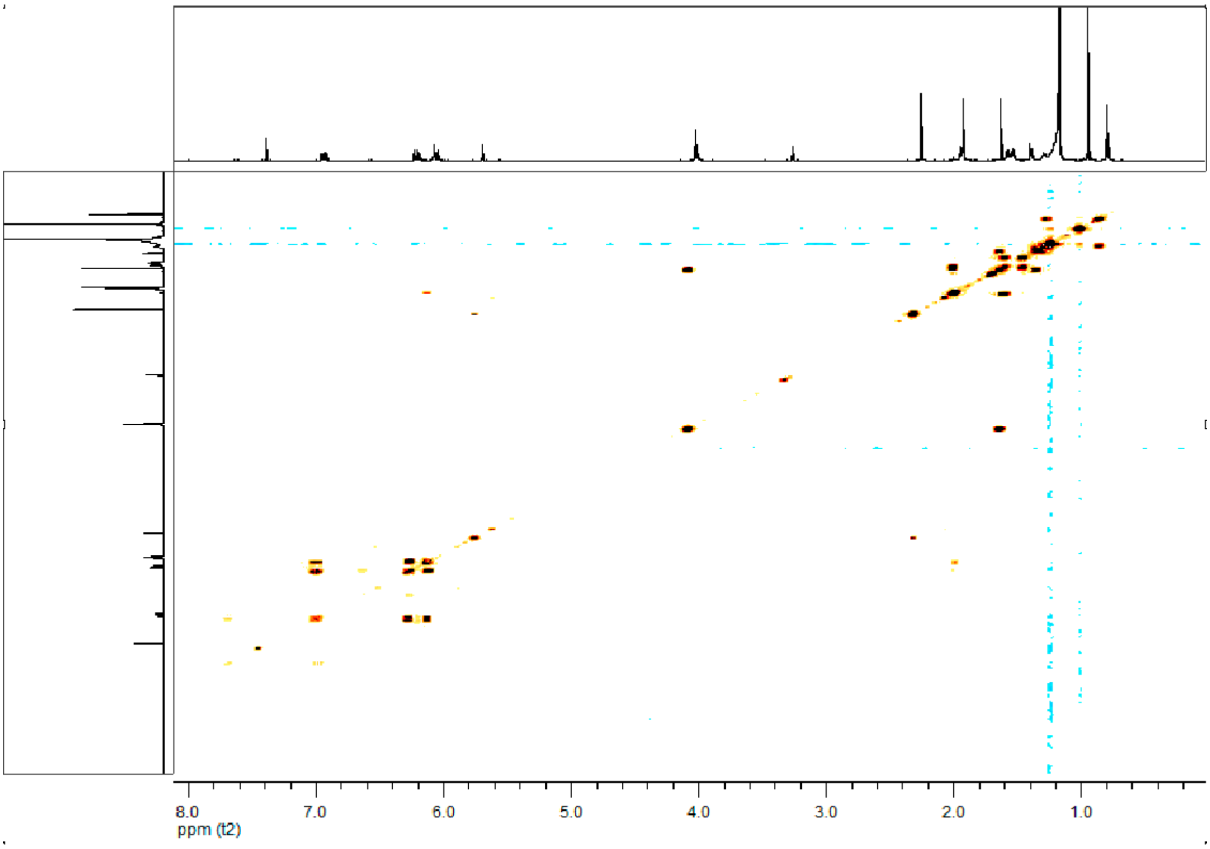

Figure S4. HSQC spectrum of ATRA-SA.

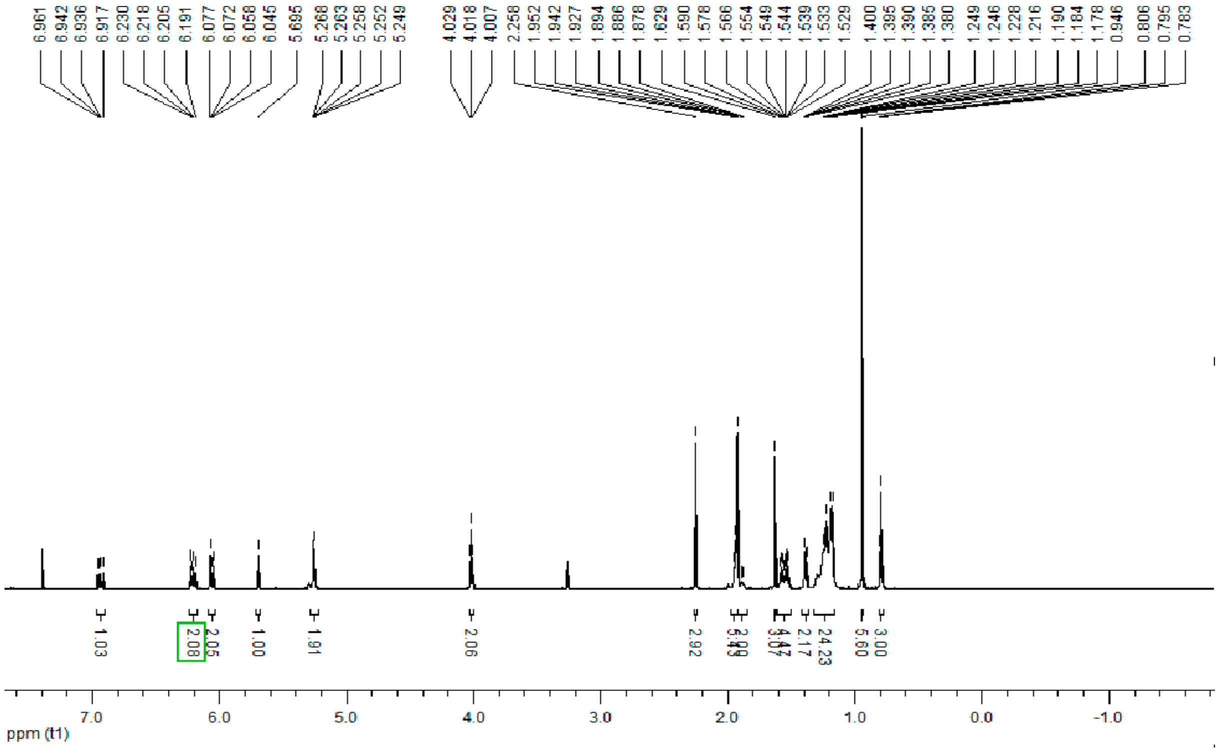

Figure S5. ^{1}H NMR spectrum (600 MHz, $CDCl_3/CD_3OD$) of ATRA-OA.

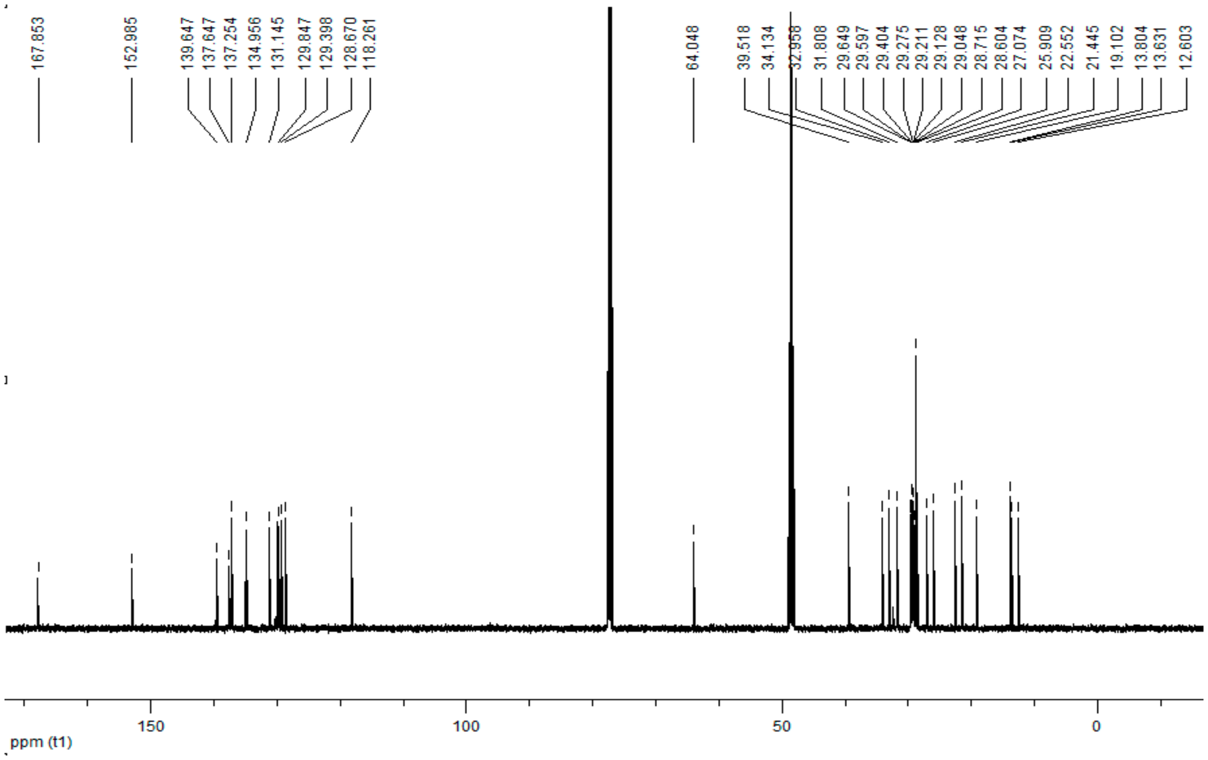

Figure S6. ^{13}C NMR spectrum (150 MHz, $CDCl_3/CD_3OD$) of ATRA-OA.

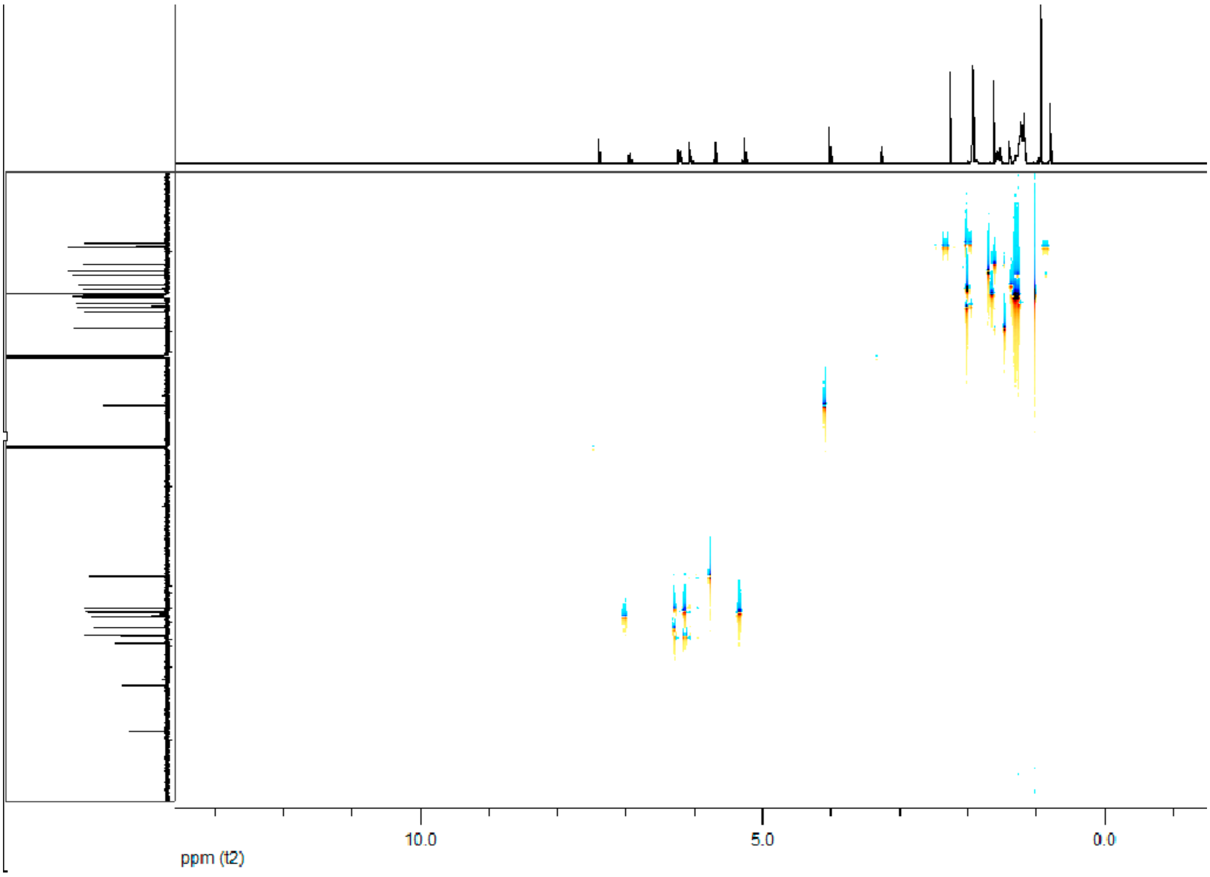

Figure S7. COSY spectrum of ATRA-OA.

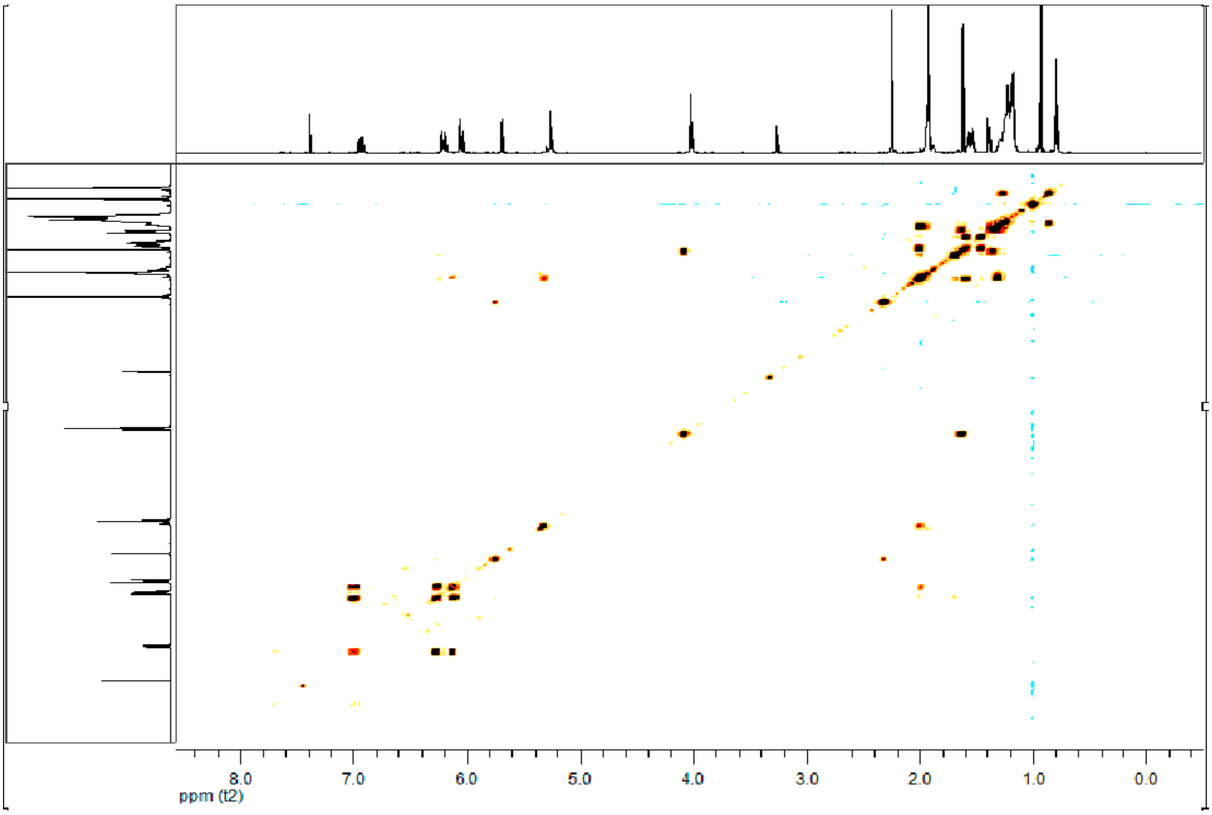

Figure S8. HSQC spectrum of ATRA-OA.

ESI-MS m/z calculated for $C_{36}H_{60}O_2$: $[M+H]^+$ 525.4672. Found 525.4686.

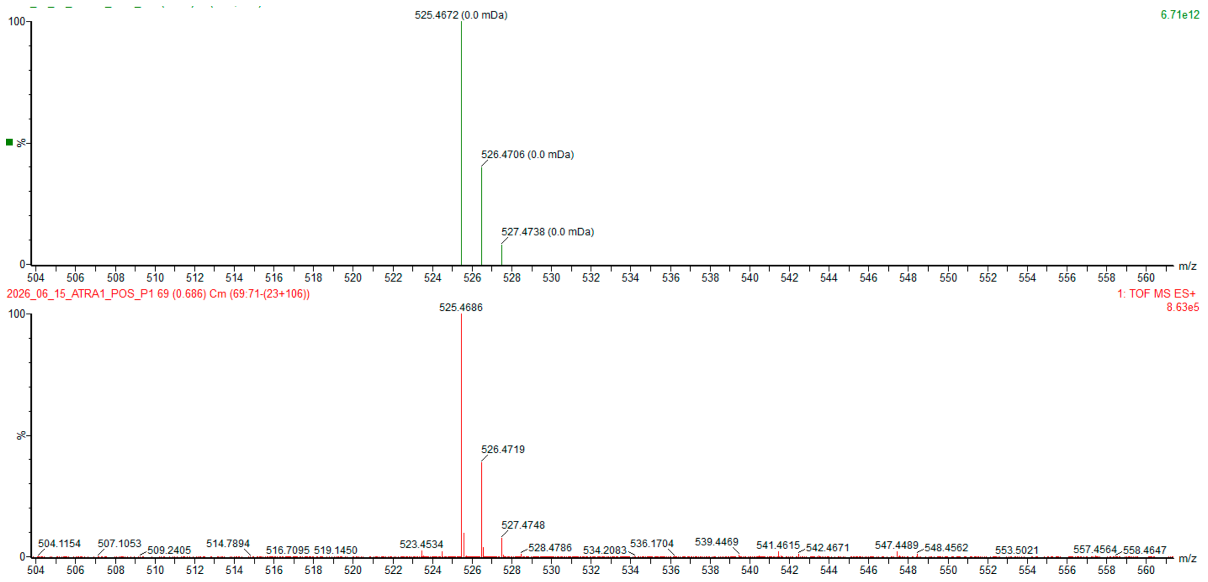

Figure S9. High-Resolution Mass Spectrometry (HRMS) Chromatogram of ATRA-CA.

ESI-MS m/z calculated for $C_{38}H_{64}O_2$: $[M+H]^+$ 553.4985. Found 553.4998.

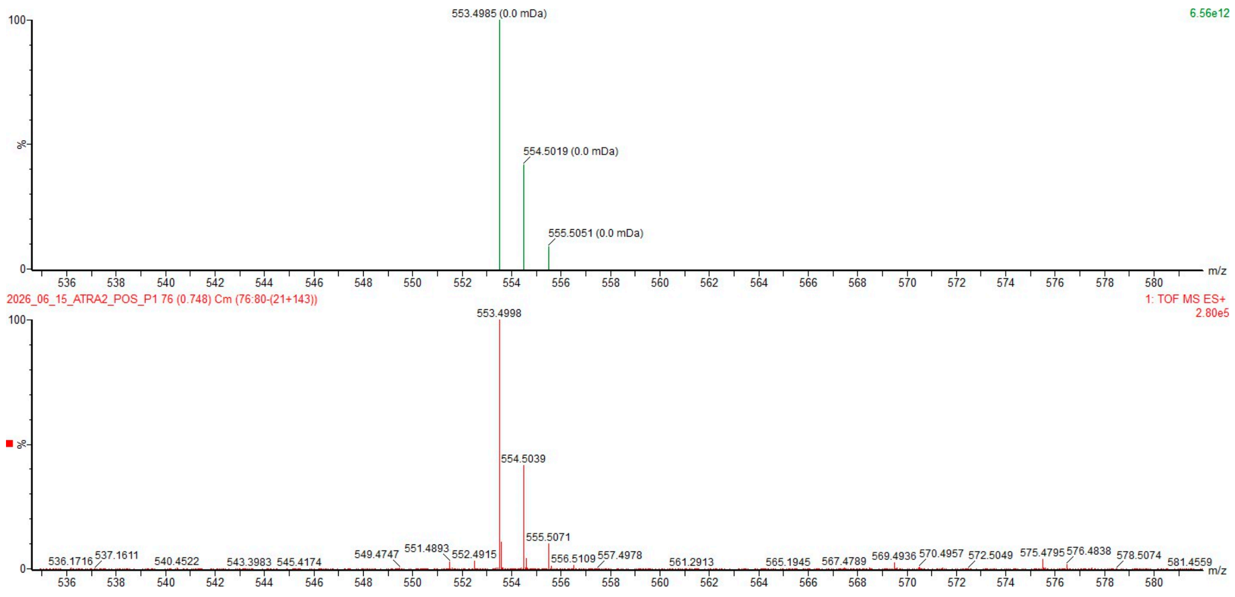

Figure S10. High-Resolution Mass Spectrometry (HRMS) Chromatogram of ATRA-SA.

ESI-MS m/z calculated for $C_{38}H_{62}O_2$: $[M+H]^+$ 551.4828. Found 551.4818.

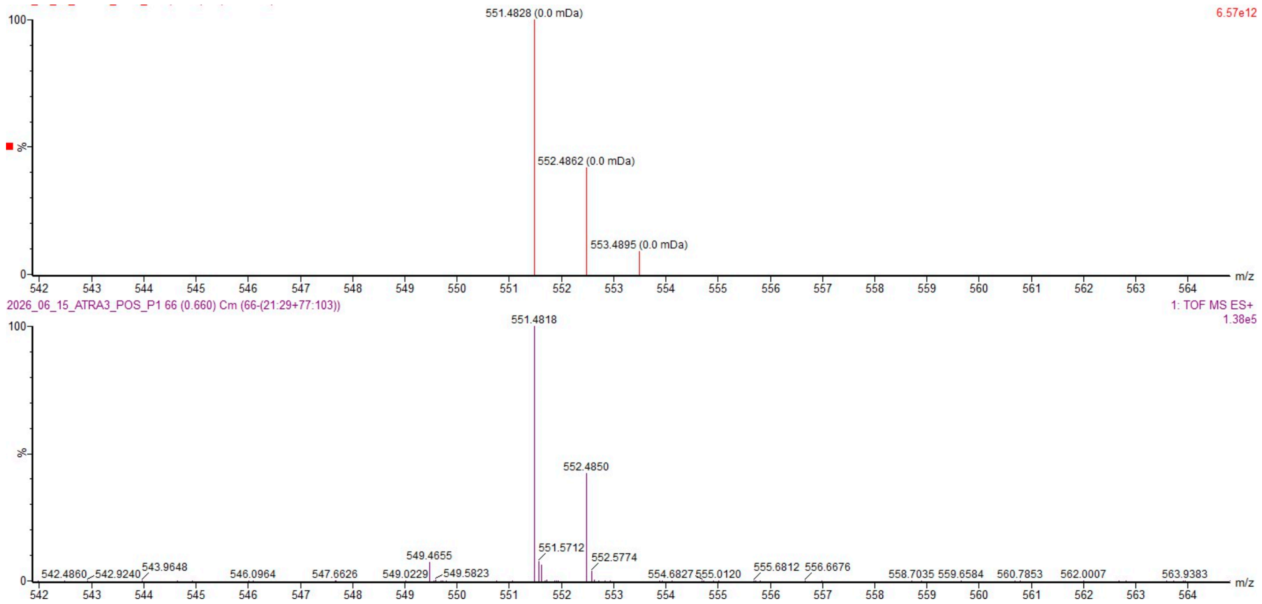

Figure S11. High-Resolution Mass Spectrometry (HRMS) Chromatogram of ATRA-OA.

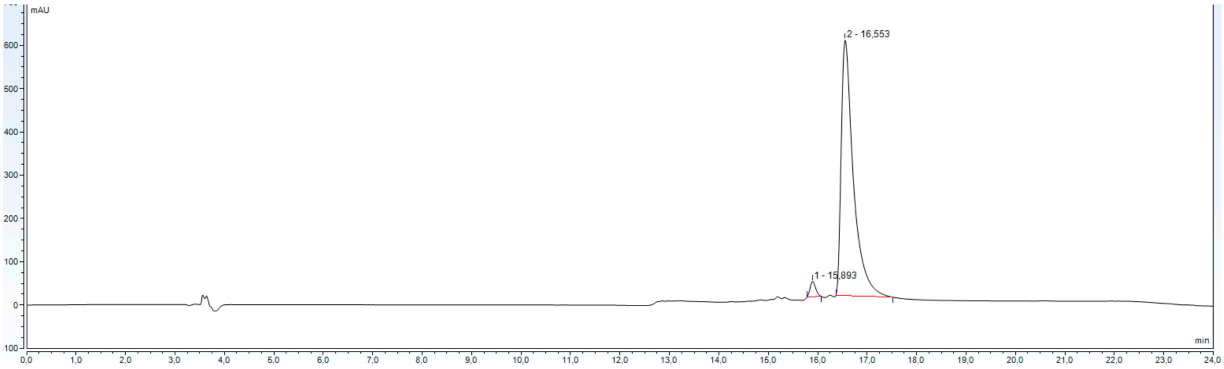

Figure S12. High-Resolution Mass Spectrometry (HRMS) Chromatogram of ATRA-CA.

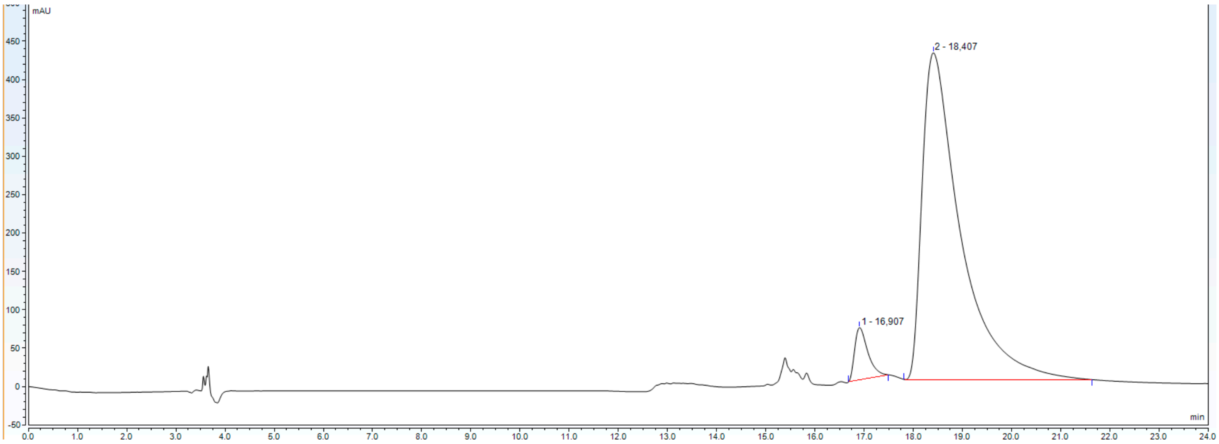

Figure S13. High-Resolution Mass Spectrometry (HRMS) Chromatogram of ATRA-SA.

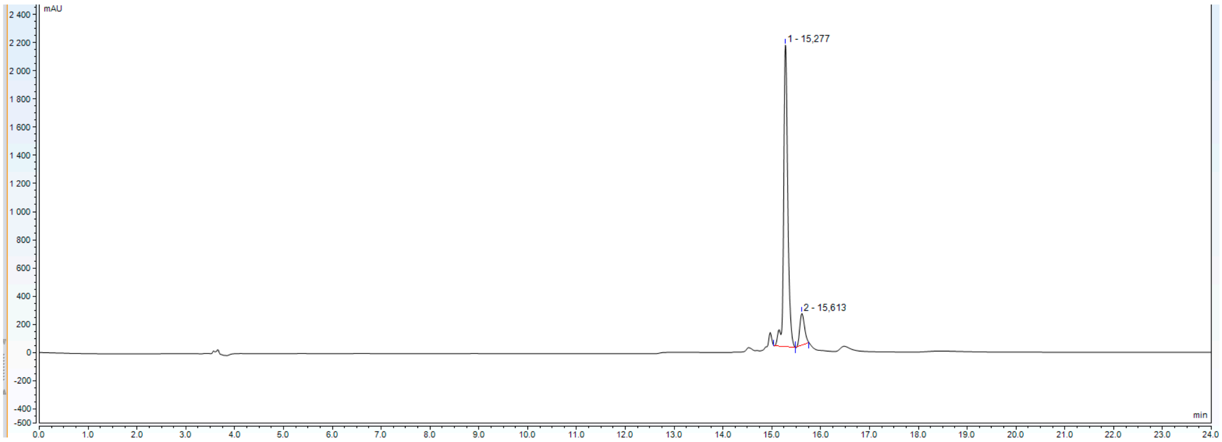

Figure S14. High-Resolution Mass Spectrometry (HRMS) Chromatogram of ATRA-OA.

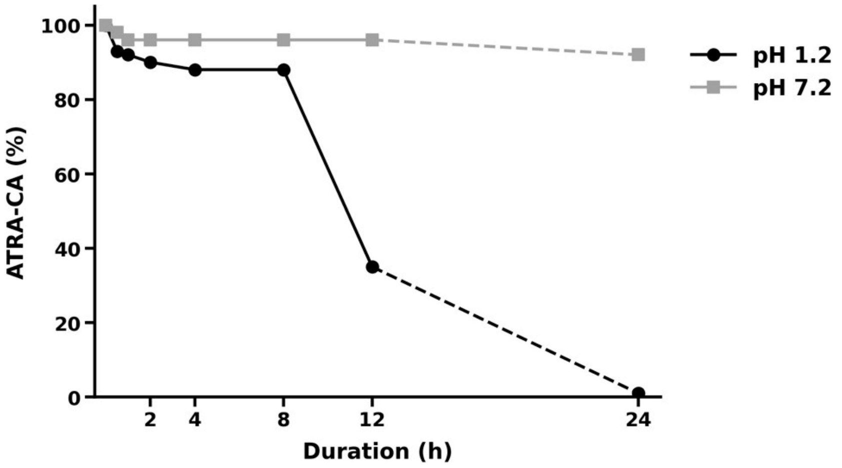

Figure S15. Time-dependent hydrolysis of ATRA-CA under acidic (pH 1.2) and physiological (pH 7.2) conditions. Dashed lines indicate intervals between measured time points and are shown only as a visual guide. No measurements were performed between 12 and 24 h; therefore, the exact time of complete hydrolysis under acidic conditions was not determined.
